# Emerging Frontiers in GLP-1 Therapeutics: A Comprehensive Evidence Base (2025)

**DOI:** 10.3390/pharmaceutics17081036

**Published:** 2025-08-09

**Authors:** Shikha Patel, Sarfaraz K. Niazi

**Affiliations:** 1 mAbtide Scientific Communications, Ahmedabad 380001, India; shikha.patel@mabtide.com; 2 College of Pharmacy, University of Illinois, Chicago, IL 60612, USA

**Keywords:** GLP-1, GLP-1 Receptor peptide Agonists, type 2 diabetes mellitus, obesity, cardiovascular disease

## Abstract

Glucagon-like peptide-1 receptor agonists (GLP-1 RAs) have evolved from glucose-lowering agents to transformative therapies across multiple organ systems. This comprehensive review synthesizes current evidence on the mechanisms, established applications, and emerging therapeutic frontiers of GLP-1 RAs. Methods: We conducted a systematic literature search of PubMed, Embase, Cochrane Library, and ClinicalTrials.gov from inception through May 2025, using controlled vocabulary and free-text terms related to GLP-1 RAs, their mechanisms, and clinical applications. Results: GLP-1 RAs demonstrate pleiotropic effects through fundamental cellular mechanisms, including enhanced mitochondrial function, anti-inflammatory actions, improved cellular quality control, and comprehensive metabolic regulation. Established applications demonstrate robust efficacy in diabetes management (HbA1c reductions of 1.5–2.0%), obesity treatment (weight loss of 7–24%), and cardiovascular protection (14–20% reduction in major adverse cardiovascular events, or MACE). Emerging applications span neurological disorders, dermatological conditions, respiratory diseases, and novel applications in addiction medicine and autoimmune disorders. Conclusions: GLP-1 RAs represent a paradigmatic shift toward multi-system therapeutic intervention, with expanding evidence supporting their role as comprehensive metabolic modulators.

## 1. Introduction

Glucagon-like peptide-1 receptor agonists (GLP-1 RAs) have emerged as one of the most transformative therapeutic classes in modern medicine. Initially developed for glycemic control in type 2 diabetes mellitus (T2DM), these medications have demonstrated unprecedented efficacy in managing obesity and protecting the cardiovascular system [1,2].

The therapeutic success of GLP-1 RAs stems from their ability to target fundamental physiological processes underlying metabolic dysfunction while maintaining a favorable safety profile relative to older antidiabetic medications [3,4]. The GLP-1 receptor, a class B G protein-coupled receptor with widespread tissue distribution including pancreatic islets, the central and peripheral nervous systems, cardiovascular tissues, kidneys, lungs, and gastrointestinal tract, mediates diverse physiological effects when activated [5,6,7].

GLP-1 RA therapeutic development has progressed through sequential generations, classified based on specific pharmacological properties including molecular structure, pharmacokinetic parameters, receptor selectivity, and clinical outcomes. This evolutionary progression reflects systematic optimization of drug efficacy, duration of action, and tolerability profiles. First-generation short-acting agents (exenatide and lixisenatide), with twice-daily or once-daily administration provided proofs of concept for incretin-based therapy but required frequent injections and offered modest efficacy. Second-generation long-acting molecules (liraglutide, dulaglutide, and semaglutide) introduced enhanced receptor engagement profiles and extended half-lives, enabling once-weekly administration while significantly improving glycemic control and weight reduction outcomes [8,9,10]. Most recently, third-generation multi-agonists (tirzepatide, retatrutide, and survodutide) targeting complementary incretin receptors have demonstrated unprecedented metabolic efficacy, representing a paradigm shift in both diabetes and obesity management [11,12].

A notable evolution in recent clinical development is the strategic use of GLP-1 RAs as a platform for combinatorial therapy, particularly in the treatment of obesity and metabolic syndrome. Unimolecular and co-formulated combination products such as CagriSema (semaglutide + cagrilintide) and tirzepatide have demonstrated synergistic effects through co-activation of GLP-1 and amylin or GIP receptors, respectively, achieving up to 22–24% weight loss in clinical trials—a magnitude previously only attainable with bariatric surgery [13,14].

## 2. Methodology

We conducted a comprehensive systematic literature review to identify and synthesize evidence on GLP-1 RAs across all therapeutic applications. Systematic searches were conducted in PubMed/MEDLINE, Embase, Cochrane Library, Web of Science, and ClinicalTrials.gov from database inception through 31 May 2025. The systematic search methodology and eligibility criteria for study selection are presented in Table 1.

Core search terms included the following:

- Primary drug terms: “Glucagon-Like Peptide-1 Receptor” OR “GLP-1 receptor agonist”;
- Specific agents: “liraglutide,” “semaglutide,” “exenatide,” “dulaglutide,” “tirzepatide,” “retatrutide”;
- Mechanism terms: “cellular signaling,” “mitochondrial function,” “autophagy,” “inflammation”;
- Clinical terms: “diabetes mellitus,” “obesity,” “cardiovascular disease,” neurological conditions.

## 3. Core Mechanisms of GLP-1 Action

### 3.1. Fundamental Cellular Signaling Pathways

**Figure 1:**
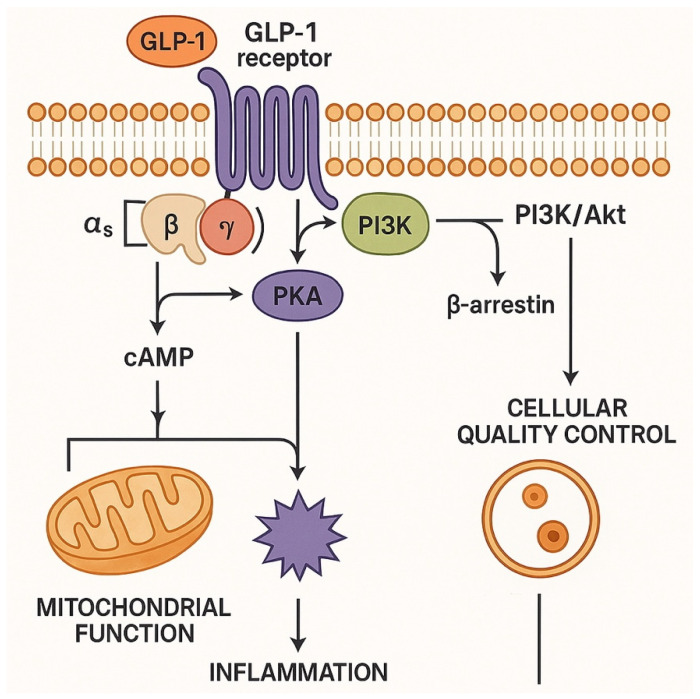
Comprehensive mechanistic diagram showing GLP-1 receptor signaling pathways including cAMP/PKA, PI3K/Akt, β-arrestin pathways, and downstream effects on mitochondrial function, inflammation, and cellular quality control.

The therapeutic efficacy of GLP-1 RAs stems from complex intracellular signaling cascades that operate across multiple organ systems through the GLP-1 receptor, a class B G protein-coupled receptor. Upon activation, the receptor initiates complex signaling networks through three primary pathways with distinct temporal and spatial characteristics (Figure 1) [15,16].

Table 2 provides a comprehensive overview of the major GLP-1 signaling pathways, including their key mediators, primary cellular effects, and clinical significance.

### 3.2. Primary cAMP/PKA Pathway

The primary signaling pathway involves Gs-mediated activation of adenylyl cyclase, leading to rapid accumulation of cyclic adenosine monophosphate (cAMP) and subsequent activation of protein kinase A (PKA). This canonical cAMP/PKA pathway phosphorylates numerous downstream targets, including the transcription factor CREB (cAMP response element-binding protein), which translocates to the nucleus and induces expression of cytoprotective genes, including brain-derived neurotrophic factor (BDNF) and B-cell lymphoma 2 (Bcl-2) [17,18]. Recent advances have revealed that GLP-1 receptor signaling creates distinct cAMP microdomains within cells, mediated by A-kinase anchoring proteins (AKAPs). This compartmentalization allows for precise spatial control of downstream responses [19,20].

### 3.3. PI3K/Akt Survival Pathway

Parallel to cAMP signaling, GLP-1 receptor activation stimulates the phosphatidylinositol 3-kinase (PI3K)/Akt pathway, a critical mediator of cell survival and metabolic regulation. Activated Akt phosphorylates multiple substrates with far-reaching consequences. Inhibition of glycogen synthase kinase-3β (GSK-3β) at Ser9 prevents tau hyperphosphorylation in neurons and enhances insulin signaling in metabolic tissues [21,22].

### 3.4. β-Arrestin-Mediated Signaling

The role of β-arrestin-2 in GLP-1 receptor signaling exhibits concentration-dependent complexity that reconciles its seemingly contradictory functions through distinct molecular mechanisms operating at different agonist concentrations. At physiological GLP-1 concentrations (<100 pM), β-arrestin-2 serves as a negative regulator, dampening insulin secretion by partially uncoupling cAMP/PKA signaling pathways, thereby providing homeostatic control under normal postprandial conditions. However, at pharmacological concentrations achieved with therapeutic GLP-1 receptor agonists (≥100 pM–10 nM), β-arrestin-2 becomes essential for sustained signaling, specifically mediating extracellular signal-regulated kinase (ERK) activation and CREB phosphorylation that promotes pancreatic β-cell survival and insulin synthesis. This apparent contradiction is mechanistically reconciled through β-arrestin-2’s dual molecular functions: classical desensitization via termination of receptor-stimulated G protein coupling (operative at all concentrations), and scaffolding function where receptor–β-arrestin-2 complexes act as signaling nodes modulating intracellular pathways (prominent at higher concentrations). The physiological relevance of this concentration-dependent signaling complexity has been demonstrated in human adipocytes, where picomolar GLP-1 promoted lipogenesis while nanomolar GLP-1 induced lipolysis. Understanding this dual role has important therapeutic implications, as G protein-biased GLP-1 receptor agonists with reduced β-arrestin recruitment show enhanced anti-hyperglycemic efficacy through avoidance of receptor desensitization, while the requirement for β-arrestin-2 in ERK/CREB activation at therapeutic concentrations explains why complete elimination of β-arrestin-2 recruitment may not be optimal for all applications. These findings highlight the importance of assessing the signaling pathways engaged by therapeutic agonists for optimal clinical outcomes. Notably, the majority of mechanistic data supporting this concentration-dependent model derives from preclinical studies using mouse pancreatic β-cells and clonal cell lines, with limited validation in human islet studies, necessitating further clinical investigation to confirm these concentration thresholds in human diabetes treatment [23,24,25,26].

### 3.5. Wnt/β-Catenin Signaling

GLP-1 receptor activation also engages the Wnt/β-catenin signaling cascade through PKA-mediated inhibition of GSK-3β. This stabilizes β-catenin, enabling nuclear translocation and activation of genes that promote neurogenesis, β-cell proliferation, and tissue regeneration [27,28]. Recent discoveries have revealed novel regulation of the Hippo pathway, with GLP-1 promoting the nuclear translocation and activation of YAP/TAZ, leading to the activation of regenerative gene programs [29].

### 3.6. Mitochondrial Enhancement and Bioenergetics

A unifying feature across GLP-1-responsive tissues is a profound enhancement of mitochondrial function and biogenesis. GLP-1 receptor activation induces expression of peroxisome proliferator-activated receptor gamma coactivator 1-alpha (PGC-1α), the master regulator of mitochondrial biogenesis, through both the cAMP/PKA/CREB and AMP-activated protein kinase (AMPK) pathways [30,31].

This leads to coordinated upregulation of nuclear respiratory factors (NRF-1 and NRF-2) and mitochondrial transcription factor A (Tfam), driving mitochondrial DNA replication and expression of respiratory chain components. GLP-1 signaling also suppresses microRNA-23a, a negative regulator of PGC-1α, while upregulating the expression of uncoupling protein 2 (UCP2) for additional protection against oxidative stress [32,33].

The functional consequences detailed in Table 3 include improved oxidative phosphorylation efficiency through the upregulation of electron transport chain complexes, particularly complex I and IV, resulting in enhanced ATP production. Mitochondrial calcium buffering capacity improves through regulation of the mitochondrial calcium uniporter (MCU), preventing calcium-induced mitochondrial dysfunction. Structural integrity is maintained through upregulation of OPA1 (optic atrophy 1), which promotes cristae organization and prevents cytochrome c release during stress conditions [34,35].

### 3.7. Anti-Inflammatory and Immunomodulatory Actions

The anti-inflammatory properties of GLP-1 RAs represent fundamental mechanisms underlying the therapeutic benefits of these agents across diverse conditions. At the molecular level, GLP-1 receptor activation potently inhibits nuclear factor kappa B (NF-κB), the master transcriptional regulator of inflammation. This occurs through cAMP-dependent PKA activation, which phosphorylates and inhibits the IκB kinase (IKK) complex [36,37].

The inflammasome, particularly the NLRP3 complex, represents another critical target. GLP-1 RAs suppress NLRP3 inflammasome activation through multiple mechanisms: inhibition of priming signals via reduced NF-κB activity, attenuation of reactive oxygen species generation through enhanced mitochondrial function, induction of autophagy that removes damaged mitochondria serving as inflammasome triggers, and direct interference with NLRP3-ASC interaction through PKA-mediated phosphorylation [38,39].

Direct immunomodulatory effects occur through GLP-1 receptors expressed on multiple immune cell populations. In macrophages, GLP-1 signaling promotes polarization from the pro-inflammatory M1 to the anti-inflammatory M2 phenotype through STAT6 activation and metabolic reprogramming [40,41,42]. T lymphocyte function is modulated through inhibition of pro-inflammatory Th17 differentiation and promotion of regulatory T cell (Treg) expansion via STAT5-dependent mechanisms [42,43].

### 3.8. Cellular Quality Control and Proteostasis

GLP-1 receptor activation enhances cellular homeostasis through comprehensive protein quality control and autophagy mechanisms. GLP-1 RAs enhance autophagy through coordinated AMPK activation and partial mTORC1 inhibition, promoting the formation of autophagosomes that engulf damaged organelles and protein aggregates [44,45]. Enhanced lysosomal biogenesis occurs through nuclear translocation of transcription factor EB (TFEB), increasing cellular degradation capacity [46,47,48].

GLP-1 signaling activates selective autophagy pathways, including mitophagy for the removal of damaged mitochondria, lipophagy for the degradation of lipid droplets, and aggrephagy for the clearance of protein aggregates. This is particularly relevant for neurodegenerative diseases where GLP-1 RAs facilitate the removal of amyloid-β, α-synuclein, and mutant huntingtin [48,49,50].

### 3.9. Pancreatic β-Cell Protection and Function

In pancreatic β-cells, GLP-1 receptor activation enhances glucose-stimulated insulin secretion by increasing intracellular cAMP production, which leads to protein kinase A phosphorylation and activation of exchange protein directly activated by cAMP (Epac). This canonical pathway promotes insulin granule exocytosis while simultaneously suppressing inappropriate glucagon secretion from α-cells [51,52]. The glucose-dependent nature of this insulinotropic action provides a significant therapeutic advantage by minimizing the risk of hypoglycemia compared to earlier antidiabetic medications. Beyond immediate glycemic control, GLP-1 signaling enhances β-cell function, promotes β-cell proliferation, and inhibits apoptosis in preclinical models [53].

GLP-1 receptor activation enhances mitochondrial biogenesis through modulation of PGC-1α, thereby improving oxidative phosphorylation, ATP production, and calcium buffering capacity [54,55]. A critical mechanism involves maintaining the mature β-cell phenotype through the regulation of key transcription factors, including pancreatic duodenal homeobox-1 (PDX1), MAF BZIP transcription factor A (MAFA), and NKX6.1 [56,57].

## 4. Established and Emerging Applications

### 4.1. Diabetes Management

#### 4.1.1. Type 2 Diabetes Mellitus

The glucose-dependent insulinotropic mechanism of GLP-1 RAs fundamentally transformed type 2 diabetes management by virtually eliminating hypoglycemia risk—a limitation that had constrained earlier antidiabetic therapies [58]. Contemporary evidence has established a clear efficacy hierarchy among agents, with dual and triple receptor agonists demonstrating superior outcomes compared to traditional GLP-1 monotherapy (Table 4).

The SURPASS-2 trial established tirzepatide’s therapeutic superiority over semaglutide 1 mg, demonstrating greater HbA1c reduction (−2.01% vs. −1.86%) and substantially superior weight loss (−10.3% vs. −6.9%) with the 15 mg dose [9]. This efficacy advantage has been confirmed through a comprehensive network meta-analysis of 76 trials encompassing 39,246 participants, which demonstrated tirzepatide 15 mg as the most efficacious agent for HbA1c reduction relative to a placebo (mean difference −2.00%; 95% CI −2.16 to −1.84) [62].

Real-world evidence from the most extensive comparative effectiveness study demonstrated that GLP-1 RA-naïve patients initiating tirzepatide achieved significantly greater HbA1c reduction compared to those initiating semaglutide (−1.3% vs. −0.9%, *p* < 0.0001), confirming that clinical trial efficacy translates to routine practice [63]. Population-level time series analysis of US adults with type 2 diabetes demonstrated that increased utilization of newer GLP-1 RAs correlates with improved population-level glycemic control [64].

Oral semaglutide represents a paradigm shift in GLP-1 therapy accessibility, with extensive real-world evidence from 19 studies confirming comparable effectiveness and tolerability to injectable formulations in routine clinical scenarios [65]. The PIONEER 6 trial demonstrated cardiovascular safety with non-inferiority to placebo for major adverse cardiovascular events [61]. A comprehensive meta-analysis, including SOUL trial data, demonstrated that long-acting GLP-1 RAs reduce the incidence of major adverse cardiovascular events (MACEs), hospitalization for heart failure, kidney events, and all-cause mortality in type 2 diabetes [66]. The SOUL trial specifically demonstrated a 14% reduction in major adverse cardiovascular events (HR 0.86; 95% CI 0.77–0.96; *p* = 0.006) among 9650 participants [67].

#### 4.1.2. Type 1 Diabetes

The application of GLP-1 RAs in type 1 diabetes exemplifies precision medicine addressing specific patient phenotypes, with concomitant obesity common among patients and only 20% achieving adequate glycemic control. A systematic review and meta-analysis of five trials involving 2445 participants demonstrated that liraglutide 1.8 mg produced a modest but clinically meaningful reduction in HbA1c (−0.24%; 95% CI −0.32 to −0.16) [68]. The 2023 updated meta-analysis confirmed therapeutic benefits primarily center on weight loss and insulin dose reduction rather than dramatic glycemic improvement [69].

### 4.2. Obesity Management

GLP-1 RAs have revolutionized obesity pharmacotherapy through comprehensive weight management mechanisms extending beyond simple appetite suppression (Table 5). The combination of delayed gastric emptying, direct hypothalamic appetite regulation, and peripheral metabolic effects achieves sustained weight reduction, approaching levels comparable to those of bariatric surgery with the most advanced agents [70,71].

A comprehensive systematic review of 41 trials involving 15,135 participants found significant weight reduction across all GLP-1 RAs (MD −5.319 kg; 95% CI −6.465 to −4.174), with additional improvements in BMI and waist circumference [76]. However, the therapeutic landscape has been transformed by multi-receptor agonists, which have achieved unprecedented weight loss levels. Network meta-analyses demonstrate a progressive efficacy gradient: GLP-1 monotherapy achieves approximately 7 kg weight reduction, dual agonists reach 11 kg, while triple agonists achieve up to 24 kg weight loss at 52 weeks [77].

Tirzepatide has established itself as highly effective, with the 15 mg dose achieving 22.5% weight loss (95% CI, −23.3 to −21.7), representing a reduction of 23.6 kg (52.0 lb) in phase 3 obesity trials [73,78]. Retatrutide represents the current efficacy frontier, demonstrating up to 24.2% weight reduction in the 12 mg group at 48 weeks, approaching bariatric surgery levels without invasive intervention [72,79,80].

### 4.3. Cardiovascular and Cardiorenal Protection

Cardiovascular protection has been demonstrated for multiple GLP-1 RAs, though with notable heterogeneity in outcomes across different agents within the class. While multiple trials have shown significant cardiovascular benefits, the evidence reveals varying magnitudes of effect and some neutral results, indicating that cardiovascular protection may not be universally consistent across all GLP-1 RAs (Table 6) [81].

**Figure 2:**
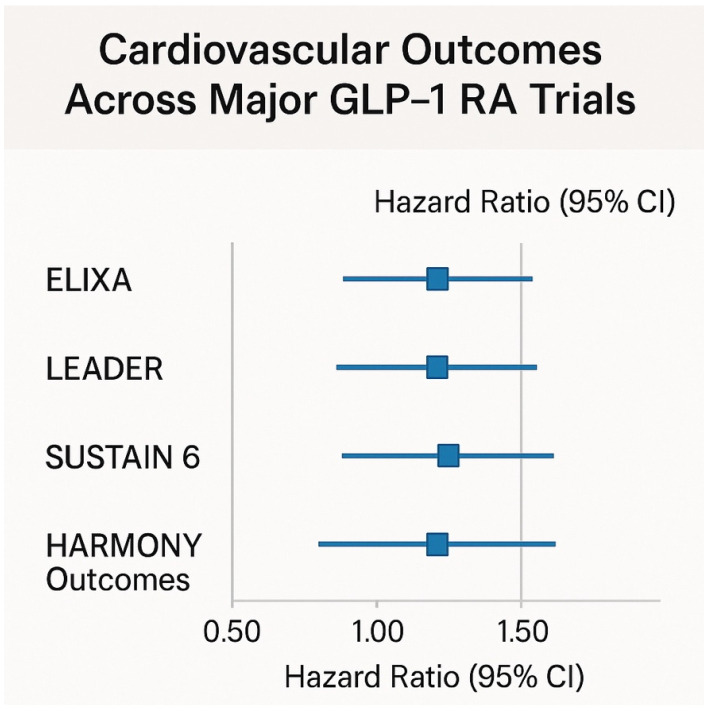
Forest plot showing cardiovascular outcomes across major GLP-1 RA trials, displaying hazard ratios and confidence intervals for MACE reduction.

As illustrated in Figure 2, pooled analyses of cardiovascular outcome trials have demonstrated that GLP-1 RAs reduce major adverse cardiovascular events (MACEs) by 14% (HR = 0.86, 95% CI 0.79–0.94) in patients with type 2 diabetes, with additional significant benefits including 13% reduction in cardiovascular death, 16% reduction in nonfatal stroke, and 10% reduction in hospitalization for heart failure [85]. However, this overall benefit reflects predominantly positive results from specific agents, with important exceptions such as lixisenatide in the ELIXA trial, which demonstrated cardiovascular safety but no significant reduction in MACEs (HR = 1.02, 95% CI 0.89–1.17) [86].

The largest meta-analysis to date, comprising 13 cardiovascular outcome trials involving 83,258 patients with and without diabetes, confirmed that GLP-1 Ras significantly reduced major adverse cardiovascular events (MACEs), all-cause mortality, cardiovascular mortality, fatal and non-fatal strokes, coronary revascularization, and composite kidney outcomes [87].

The landmark SELECT trial significantly expanded the therapeutic paradigm by demonstrating a 20% relative risk reduction in three-point major adverse cardiovascular events (MACEs) with semaglutide 2.4 mg in 17,604 patients with obesity but without diabetes, leading to FDA approval for cardiovascular risk reduction in March 2024 [82]. This groundbreaking finding extends cardiovascular benefits beyond the diabetic population to individuals with obesity alone.

Individual trials have established agent-specific evidence profiles, revealing differential cardiovascular efficacy within the GLP-1 RA class. The REWIND trial demonstrated dulaglutide’s effectiveness in reducing the primary composite endpoint of non-fatal myocardial infarction, non-fatal stroke, or death from cardiovascular causes by 12% [59]. The SURPASS-CVOT trial is an ongoing phase 3 cardiovascular outcomes trial designed to evaluate the non-inferiority and potential superiority of tirzepatide compared to dulaglutide in cardiovascular outcomes [88,89].

#### Heart Failure Benefits

The most significant recent advancement has been the demonstration of profound benefits in heart failure with preserved ejection fraction (HFpEF) across multiple pivotal trials, establishing GLP-1 RAs as transformative therapy for obesity-related heart failure (Table 7).

The landmark SUMMIT trial with tirzepatide has provided the most compelling evidence to date for heart failure benefits with incretin-based therapy. In 731 patients with HFpEF and obesity, tirzepatide demonstrated a 38% reduction in the composite endpoint of cardiovascular death or worsening heart failure events (HR 0.62, 95% CI 0.41–0.95), representing one of the most significant treatment effects ever observed in HFpEF [90].

The STEP-HFpEF program established comprehensive benefits in heart failure with preserved ejection fraction across diverse populations. Clinical trials have demonstrated the efficacy of semaglutide in HFpEF, with significant benefits in symptoms and quality of life measures [91]. Pooled analyses across the SELECT, FLOW, STEP-HFpEF, and STEP-HFpEF DM trials confirmed a consistent reduction in heart failure events among 3743 participants with HFpEF [93].

### 4.4. Renal Protection

Recent evidence suggests that GLP-1 RAs exhibit significant nephroprotective effects, extending beyond their cardiovascular benefits. A comprehensive meta-analysis of 11 trials involving 85,373 participants demonstrated that GLP-1 RAs reduced composite kidney outcomes by 18% (HR 0.82, 95% CI 0.73–0.93), kidney failure by 16% (HR 0.84, 0.72–0.99), MACEs by 13% (HR 0.87, 0.81–0.93), and all-cause death by 12% (HR 0.88, 0.83–0.93) in participants with type 2 diabetes [94,95].

The pivotal FLOW trial demonstrated semaglutide’s nephroprotective effects, with a 24% reduction in the primary composite renal outcome (HR 0.76; 95% CI, 0.66 to 0.88; *p* = 0.0003) in 3533 patients with type 2 diabetes and chronic kidney disease, over a median follow-up of 3.4 years [96,97]. The trial was terminated early due to overwhelming efficacy, leading to regulatory approval for the prevention of chronic kidney disease progression in people with type 2 diabetes and early kidney disease evidence.

### 4.5. Metabolic Dysfunction-Associated Steatotic Liver Disease

Non-alcoholic steatohepatitis (NASH) and fatty liver disease, now termed metabolic dysfunction-associated steatohepatitis (MASH) and steatotic liver disease (MASLD), represent significant therapeutic targets where GLP-1 RAs demonstrate substantial therapeutic potential, as evidenced by multiple clinical trials (Table 8) [98,99].

GLP-1 RAs ameliorate hepatic steatosis through improved insulin sensitivity, direct suppression of hepatic de novo lipogenesis through AMPK activation and SREBP-1c inhibition, and enhanced fatty acid oxidation through PPARα upregulation. AMPK plays a crucial role in the development and progression of MASLD, representing a promising therapeutic target through its diverse signaling input and output networks [105,106].

The pivotal phase 3 ESSENCE trial with semaglutide 2.4 mg weekly demonstrated superior efficacy across both primary endpoints at 72 weeks. For the first primary endpoint, 62.9% of participants treated with semaglutide achieved resolution of steatohepatitis, with no worsening of liver fibrosis, compared to 34.1% on placebo (estimated difference in responder proportions [EDP] of 28.7%; 95% CI, 21.1 to 36.2; *p* < 0.001) [100].

Tirzepatide (5–15 mg weekly) evaluated over 52 weeks in the SYNERGY-NASH trial with participants having MASH and stage F2 or F3 fibrosis showed MASH resolution without worsening fibrosis in 44%, 56%, and 62% of participants on 5 mg, 10 mg, and 15 mg doses, respectively, compared to 10% with placebo [102,107].

Survodutide, a dual glucagon/GLP-1 RA, has demonstrated exceptional efficacy in phase 2 trials. Studies in 293 subjects with biopsy-confirmed MASH showed improved MASH without worsening fibrosis in 47%, 62%, and 43% of participants receiving 2.4 mg, 4.8 mg, and 6.0 mg doses, respectively, versus 14% with placebo [103,108].

## 5. Neurological Disorders

Emerging clinical evidence reveals significant therapeutic potential for GLP-1 RAs in treating neurological disorders (Table 9).

### 5.1. Alzheimer’s Disease

In Alzheimer’s disease models, GLP-1 RAs demonstrate multiple beneficial mechanisms, including reduced amyloid-β and tau pathology through enhanced clearance mechanisms, decreased neuroinflammation by modulating microglial activation states, and enhanced synaptic plasticity through improved long-term potentiation and dendritic spine density [35,109].

**Table 9:** GLP-1 RAs in Neurological Disorders.

Condition	Agent	Study	Primary Finding	Status	Reference
Alzheimer’s Disease	Liraglutide	ELAD	50% less brain volume loss	Completed	[110]
	Oral Semaglutide	EVOKE/EVOKE+	CDR-Sum of Boxes	Ongoing	[111,112,113]
Parkinson’s Disease	Exenatide	EXENATIDE-PD3	No disease modification	Completed	[114]
	Lixisenatide	LixiPark	Slower motor progression	Completed	[115]
	Semaglutide	Oslo University	Motor symptoms	Ongoing	[116,117]

The ELAD trial, a randomized, double-blind, placebo-controlled study of 204 patients with mild Alzheimer’s disease, demonstrated that liraglutide treatment resulted in nearly 50% less brain volume loss in frontal, temporal, parietal, and total gray matter regions compared to placebo, suggesting significant neuroprotective effects [110,118]. Clinical evaluation continues with the EVOKE and EVOKE+ trials, the first large-scale phase 3 studies evaluating oral semaglutide in people aged 55–85 years with mild cognitive impairment or mild dementia and evidence of Alzheimer’s pathology [112,118,119].

### 5.2. Parkinson’s Disease

GLP-1 RAs exhibit neuroprotective effects by reducing α-synuclein aggregation through enhanced chaperone-mediated autophagy, attenuating neuroinflammation via microglial modulation, and promoting dopaminergic neuron survival through anti-apoptotic signaling and mitochondrial protection [22,35,120].

Clinical investigations have yielded mixed results, with the LixiPark trial demonstrating potential disease-modifying effects in patients with early Parkinson’s disease, showing slower motor symptom progression compared to the placebo group [115]. However, the most significant phase 3 trial (EXENATIDE-PD3), which used exenatide once weekly over 96 weeks in 194 people with Parkinson’s disease, found no evidence to support exenatide as a disease-modifying treatment [121].

Real-world data from a comprehensive US Veterans Affairs Health Care system study of 215,970 individuals with type 2 diabetes revealed that GLP-1 medications were associated with reduced rates of neurocognitive disorders, including Alzheimer’s disease (HR 0.88) and dementia (HR 0.92), as well as decreased risks of substance use disorders and seizures [122].

## 6. Reproductive Health Applications

Polycystic ovary syndrome (PCOS) represents a metabolic-reproductive disorder where GLP-1 RAs show therapeutic potential by addressing core pathophysiological mechanisms of insulin resistance and compensatory hyperinsulinemia that exacerbate ovarian androgen production [123,124]. GLP-1 RAs enhance insulin sensitivity by increasing signaling in skeletal muscle, liver, and adipose tissue, reduce hyperinsulinemia through improved pancreatic β-cell function, and facilitate weight loss by addressing adipose tissue dysfunction [125,126,127].

Clinical evidence from multiple randomized controlled trials and meta-analyses demonstrates significant therapeutic benefits of GLP-1 RAs in women with PCOS. A meta-analysis by Han et al. of eight RCTs involving 375 women showed that compared with metformin, GLP-1 RAs were more effective in improving insulin sensitivity (SMD −0.40, 95% CI −0.74 to −0.06, *p* = 0.02) and reducing body mass index (SMD −1.02, 95% CI −1.85 to −0.19, *p* = 0.02) [128]. A recent meta-analysis by Morais et al. of four RCTs with 176 participants demonstrated that GLP1-RAs significantly reduced waist circumference (MD: −5.16 cm), BMI (MD: −2.42 kg/m^2^), serum triglycerides (MD: −0.20), and total testosterone levels (MD: −1.33) compared to placebo [129].

Reproductive outcomes showed meaningful improvements, with Zhou et al.’s meta-analysis revealing that GLP1RAs improved natural pregnancy rate (RR: 1.72, 95% CI 1.22 to 2.43, *p* = 0.002) and menstrual regularity (SMD: 1.72, 95% CI 0.60 to 2.85, *p* < 0.001) [130].

## 7. Dermatological Applications

Inflammatory skin disorders represent promising therapeutic targets for GLP-1RAs, with psoriasis receiving particular attention due to its strong associations with metabolic syndrome. GLP-1 receptors are extensively expressed on keratinocytes, dermal fibroblasts, and skin-resident immune cells, providing a mechanistic rationale for therapeutic intervention [131]. Table 10 highlights key dermatological benefits of GLP-1 RAs.

The primary anti-inflammatory mechanisms include the inhibition of JAK-STAT and NF-κB signaling pathways in keratinocytes, enhanced keratinocyte barrier function through increased expression of tight junction proteins, reduced Th17 polarization that drives psoriatic pathology, and reduced oxidative stress through enhanced expression of antioxidant enzymes [131,139,140]. Additionally, GLP-1RAs inhibit key inflammatory mediators, including tumor necrosis factor-alpha (TNF-α), nuclear factor-kappa B (NF-κB), interleukin (IL)−23, IL−17, and IL−22 [141,142,143].

Clinical evidence demonstrates significant therapeutic benefits of GLP-1RAs in the management of psoriasis. A comprehensive systematic review and meta-analysis published in 2024 demonstrated that treatment with GLP-1 RAs results in significant reductions in disease severity among patients with psoriasis, irrespective of whether they have diabetes mellitus [134,144].

## 8. Respiratory Applications

Chronic respiratory conditions, particularly asthma and chronic obstructive pulmonary disease, represent significant emerging applications supported by GLP-1 receptor expression throughout the bronchial epithelium, airway smooth muscle, and pulmonary immune cells (Table 11) [145,146].

A comprehensive meta-analysis of 28 randomized controlled trials involving 77,485 participants demonstrated that GLP-1 RAs were associated with a 14% lower risk of respiratory disease compared to controls (relative risk, 0.86; 95% confidence interval, 0.81–0.93; *p* < 0.0001). Among individual agents, semaglutide showed the most potent protective effect (RR 0.82, 95% CI 0.68–0.97, *p* = 0.02), followed by liraglutide (RR 0.86, 95% CI 0.75–0.98, *p* = 0.03), and dulaglutide (RR 0.82, 95% CI 0.70–0.96, *p* = 0.02) [147,153].

The most comprehensive evidence to date comes from a landmark 2025 study by See et al., which utilized the TriNetX Analytics Network database to analyze 6898 patients with COPD and type 2 diabetes who received single-inhaler triple therapy (SITT). After propensity score matching, 1751 GLP-1 analog users showed significant improvements compared to 1751 DPP-4 inhibitor users across multiple outcomes. GLP-1 analog users had an 18% lower risk of COPD exacerbation (hazard ratio 0.82, 95% CI 0.71–0.94, *p* = 0.003), a 28% reduced risk of pneumonia (HR 0.72, 95% CI 0.61–0.85, *p* < 0.001), a 34% reduced risk of oxygen dependence (HR 0.66, 95% CI 0.47–0.91, *p* = 0.010), and a 40% decreased risk of all-cause mortality (HR 0.60, 95% CI 0.47–0.77, *p* < 0.001) [149].

## 9. Sleep Disorders

Obstructive sleep apnea (OSA) represents a significant clinical breakthrough for GLP-1 RAs in sleep medicine, culminating in the historic FDA approval of tirzepatide (Zepbound) on December 20, 2024, as the first medication specifically indicated for moderate-to-severe OSA in adults with obesity [154]. Current evidence supporting GLP-1 RAs applications in sleep disorders is outlined in Table 12.

This approval was based on the pivotal SURMOUNT-OSA phase 3 trials, which involved 469 participants and demonstrated unprecedented efficacy. Tirzepatide achieved up to a 62.8% reduction in the apnea–hypopnea index (AHI), equivalent to 25–30 fewer events per hour compared to placebo. In patients not using positive airway pressure therapy, tirzepatide reduced the AHI by 27.4 events per hour, compared to 4.8 with placebo (55% reduction). Remarkably, 42–50% of participants achieved clinical remission or mild OSA severity compared to only 14–16% with a placebo [155,156,161].

The therapeutic mechanisms underlying OSA improvement extend beyond weight loss alone, encompassing both weight-dependent and weight-independent pathways. Weight-dependent mechanisms include reduced upper airway collapsibility through decreased parapharyngeal fat deposition [162,163]. Weight-independent mechanisms involve enhanced upper airway dilator muscle function, reduced systemic inflammation contributing to airway edema, enhanced central respiratory drive through brainstem modulation, and improved sleep architecture through enhanced slow-wave sleep duration [164,165].

## 10. Musculoskeletal Applications

Musculoskeletal applications of GLP-1 RAs gained substantial momentum in 2024–2025, particularly for the management of osteoarthritis and sarcopenia, with systematic reviews identifying consistent signals supporting favorable structural, protective, immunomodulatory, and analgesic effects [166].

The STEP-9 trial, which examined semaglutide in 407 patients with obesity and knee osteoarthritis, documented meaningful improvements in pain and physical function scores associated with a 13.7% weight loss. From baseline to week 68, the mean change in knee pain, assessed using the WOMAC pain score, was a reduction of 41.7 points for semaglutide and a decrease of 27.5 points for placebo, with an estimated treatment difference of 14.1 points, which was statistically significant [167].

Groundbreaking research published in *Science* in 2024 revealed a novel gut–joint axis mechanism for osteoarthritis treatment via GLP-1-mediated pathways. This research identified altered microbial bile acid metabolism, characterized by reduced glycoursodeoxycholic acid (GUDCA), in patients with osteoarthritis. It demonstrated that GLP-1 RAs can modulate intestinal farnesoid X receptor (FXR) signaling to improve joint health [168].

For sarcopenia and age-related muscle dysfunction, GLP-1 RAs preserve muscle health through prevention of catabolic muscle protein breakdown, enhanced insulin and amino acid-stimulated muscle protein synthesis through improved mTOR pathway activation, reduced skeletal muscle inflammation and fibrosis, and enhanced mitochondrial biogenesis improving muscle metabolic efficiency [169,170,171,172].

However, concerns about muscle mass loss with GLP-1 RAs have been raised by recent research. Studies suggest muscle loss with these medications (as indicated by decreases in fat-free mass) ranges from 25% to 39% of the total weight lost over 36–72 weeks [173,174]. In the STEP-1 trial of semaglutide, lean mass was reduced by 6.92 kg or 13.2%, with a weight reduction of 15.3 kg or 14.9%, resulting in a fraction of weight lost from lean mass of 45.2%. Similarly, in the SURMOUNT-1 trial of tirzepatide, lean mass was reduced by −5.67 kg or −10.9% from baseline, with a weight reduction of −22.1 kg or −20.9% (with the highest dose), yielding a fraction of weight lost from lean mass of 25.7% [175].

## 11. Pediatric Applications

The application of GLP-1 RAs in pediatric populations has experienced dramatic growth, with prescription rates for adolescents aged 12–17 years increasing by nearly 600% from 2020 to 2024 [176]. The 2024 average semaglutide prescription rate among adolescents with obesity was 23.7-fold higher than liraglutide prescription rates [177].

The landmark STEP TEENS trial demonstrated the remarkable efficacy of semaglutide in adolescents aged 12–17 years with obesity. After 68 weeks of treatment, participants receiving semaglutide 2.4 mg weekly achieved a mean BMI reduction of 16.1%, compared to 0.6% with placebo. Additionally, 73% of participants achieved a weight loss of ≥5%, compared to 18% with placebo [178].

The safety profile in adolescents appears comparable to that in adults, with gastrointestinal symptoms predominating as transient adverse events. Importantly, no significant adverse effects on linear growth, pubertal development, or bone health were observed during treatment periods [179].

Current FDA-approved GLP-1 RAs for adolescents include semaglutide (Wegovy), approved in December 2022, and liraglutide (Saxenda), approved in December 2020 for individuals aged 12 and older [180]. Tirzepatide (Mounjaro/Zepbound) remains approved exclusively for adults aged 18 years and older, as pediatric safety and efficacy data are pending from ongoing phase 3 trials in adolescents with type 2 diabetes [181]. The American Academy of Pediatrics updated its guidelines in 2023 to recommend offering medications to patients 12 years and older with obesity alongside lifestyle treatment [182].

## 12. Hematological Applications

The most significant development in hematological applications has been in the management of sickle cell disease. A 2024 retrospective analysis examined adults with sickle cell disease treated with GLP-1 RAs, demonstrating improvement in estimated glomerular filtration rate (eGFR) slope in 80% of patients receiving GLP-1 RA treatment [183].

Among five patients treated with GLP-1 RAs (liraglutide or dulaglutide) for a median duration of 21.8 months, serum glucose concentrations improved from a median of 213 mg/dL to 152 mg/dL during treatment. The eGFR slope improved from a median of −2.37 mL/min/1.73 m^2^ per year before treatment to +0.49 mL/min/1.73 m^2^ per year during treatment, indicating significant renoprotective benefits [183].

Limited evidence from a large retrospective cohort study of 2025 observed statistical association between GLP-1 RA use and a reduced incidence of hematologic cancers, including leukemia and myelodysplastic syndromes, in patients with type 2 diabetes. However, these findings represent observational correlations rather than established causality and require confirmation through randomized controlled trials [184].

## 13. Autoimmune and Inflammatory Disorders

### 13.1. Autoimmune Thyroid Disorders

Current evidence for GLP-1 RAs in autoimmune thyroid disorders remains primarily mechanistic with limited clinical data, though the theoretical rationale is compelling. GLP-1 RAs demonstrate well-established anti-inflammatory and metabolic regulatory properties that may benefit autoimmune thyroid disorders through multiple mechanisms. These include decreased systemic inflammatory cytokines (TNF-α, IL-1β, and IL-6), reduced thyroid-infiltrating inflammatory cells through PI3K-Akt pathway modulation, improved metabolic function, and reduced oxidative damage through increased antioxidant capacity [185,186,187].

However, thyroid cancer risk remains a complex and evolving safety consideration. Meanwhile, large-scale prospective cohort studies from 2024 to 2025, including a multisite analysis of 98,147 GLP-1RA users across six countries, show no evidence of increased thyroid cancer risk [188]. However, pharmacovigilance data presents a contrasting signal. FDA Adverse Event Reporting System analysis revealed significant disproportionate reporting of thyroid cancer with GLP-1 RAs, with reporting odds ratios ranging from 2.09 (tirzepatide) to 15.59 (liraglutide) [189]. Additionally, temporal detection patterns emerge, as US claims data revealed increased thyroid cancer diagnoses within the first year of GLP-1RA initiation, potentially reflecting enhanced medical surveillance rather than true carcinogenesis. Additionally, in patients with pre-existing thyroid nodules using GLP-1 analogs, approximately 9% developed thyroid cancer during follow-up, highlighting the importance of enhanced surveillance in this higher-risk population [190].

### 13.2. Inflammatory Bowel Disease

Inflammatory bowel disease represents a promising therapeutic target with emerging evidence supporting beneficial effects in both Crohn’s disease and ulcerative colitis. A 2024 retrospective analysis of 244 IBD patients treated with GLP-1 RAs demonstrated significant weight loss (mean 5% reduction at 12 weeks) and, importantly, a decrease in C-reactive protein levels, suggesting potential anti-inflammatory effects [191,192].

## 14. Substance Abuse Disorders and Addiction Treatment

The role of GLP-1 RAs in substance use disorders has gained substantial momentum in 2024–2025, with multiple large-scale observational studies and ongoing clinical trials. GLP-1 receptors are extensively expressed in brain reward circuits, particularly mesolimbic and mesocortical pathways involved in addictive behaviors [193].

A pivotal study analyzing 503,747 patients with opioid use disorder and 817,309 patients with alcohol use disorder found that prescriptions of GLP-1 RAs were associated with significantly lower rates of opioid overdose and alcohol intoxication [194]. Clinical evidence includes studies documenting reduced alcohol consumption, fewer drinks per day, and decreased alcohol craving with semaglutide treatment. Observational data from Sweden revealed that semaglutide and liraglutide were associated with decreased hospitalization risk due to alcohol use disorder in people with type 2 diabetes and obesity [195,196].

## 15. Psychiatric Disorders

The bidirectional relationship between metabolic disorders and mental health conditions has prompted the investigation of GLP-1 RAs in psychiatric applications. GLP-1 receptors are expressed in mood regulation brain regions, including the amygdala, hippocampus, prefrontal cortex, ventral tegmental area (VTA), and nucleus accumbens (NAc), where signaling modulates monoaminergic neurotransmission and hypothalamic–pituitary–adrenal axis function [197,198].

Systematic reviews and meta-analyses have provided compelling evidence for the antidepressant effects of GLP-1 RAs. A recent meta-analysis of six studies involving 2071 participants found that GLP-1 RA treatment significantly reduced depression rating scale scores compared to control treatments (standardized mean difference, −0.12; 95% CI, −0.21 to −0.03) [199].

Healthcare resource utilization data from 774,968 adults with type 2 diabetes revealed that GLP-1 RA use was associated with significant reductions in outpatient hospital visits for depression (IRR: 0.96; 95% CI: 0.95–0.98) and office visits for both depression (IRR: 0.87; 95% CI: 0.82–0.92) and anxiety (IRR: 0.85; 95% CI: 0.81–0.90) compared to DPP-4 inhibitors [200].

## 16. Safety Considerations

Comprehensive safety considerations for GLP-1 RA therapy are systematically presented in Table 13.

While the safety profile remains generally favorable with predominantly transient gastrointestinal effects, emerging considerations include potential thyroid cancer risk, perioperative complications, psychiatric effects, and significant muscle mass loss with rapid weight reduction. These necessitate careful patient selection, comprehensive monitoring, and risk mitigation strategies [4,201,202].

The SUSTAIN-6 and PIONEER 6 trials showed increased diabetic retinopathy complications with semaglutide, particularly in patients with existing retinopathy (HR 1.76; 95% CI 1.11–2.78; *p* = 0.02) [61,83]. This risk appears linked to rapid glycemic improvement, consistent with established mechanisms observed with intensive insulin therapy. However, retrospective real-world evidence observed the progression rates from severe non-proliferative to proliferative diabetic retinopathy lower than anticipated over a mean follow-up of 2.9 years [203]. The ongoing FOCUS trial (NCT03811561), a dedicated post-authorization safety study utilizing standardized ophthalmic assessments, is expected to provide definitive evidence regarding long-term safety data by 2027.

## 17. Future Directions and Research Priorities

The rapid evolution of GLP-1 RAs from single-indication diabetes medications to multi-system therapeutic platforms creates unprecedented opportunities for continued innovation and clinical advancement. Future research priorities must address fundamental mechanistic questions, explore novel therapeutic applications, overcome current limitations, and ensure equitable access to these transformative therapies. Strategic research priorities encompass mechanistic insights, expanded clinical applications, technological advances, and implementation strategies, each with distinct timelines and anticipated population health impacts are presented in Table 14.

Mechanistic research priorities focus on elucidating tissue-specific and context-dependent GLP-1 receptor signaling pathways, aiming to facilitate the development of next-generation therapeutics with enhanced efficacy and safety profiles. Understanding how β-arrestin-mediated pathways contribute to therapeutic effects versus adverse events could facilitate the development of biased agonists with enhanced therapeutic windows.

Clinical development priorities encompass expansion into novel therapeutic areas where mechanistic rationale supports potential benefits, with neurological disorders representing a particularly promising frontier. Completion of ongoing Alzheimer’s trials will provide definitive evidence for disease-modifying potential, while future studies should explore applications in multiple sclerosis, amyotrophic lateral sclerosis, and traumatic brain injury.

## 18. Conclusions

GLP-1 RAs represent a paradigmatic transformation in modern therapeutics, evolving from glucose-lowering agents to comprehensive multi-system interventions that address fundamental pathophysiological processes across diverse medical conditions. This extensive review demonstrates how GLP-1 RAs have transcended traditional therapeutic boundaries by modulating core cellular mechanisms, including mitochondrial enhancement, anti-inflammatory actions, autophagy-mediated quality control, and neuroprotective pathways that underlie multiple disease states.

The clinical evidence establishes unprecedented therapeutic efficacy across established indications, with newer multi-receptor agonists achieving HbA1c reductions exceeding 2.0% in diabetes, weight loss approaching 24% in obesity that rivals bariatric surgery outcomes, and cardiovascular protection demonstrating consistent 14–20% MACE reduction with emerging benefits in heart failure with preserved ejection fraction. The expansion into novel applications spanning neurological disorders, sleep medicine, dermatology, respiratory diseases, and substance use disorders reflects the broad therapeutic potential of targeting fundamental biological pathways that contribute to diverse pathological processes.

The mechanistic foundation underlying this therapeutic versatility stems from the widespread distribution of the GLP-1 receptor and its pleiotropic signaling effects, which create a unified framework for understanding clinical benefits across seemingly disparate conditions. The progression from single receptor agonists to dual and triple receptor targeting demonstrates how mechanistic insights can drive therapeutic innovation, with combination approaches achieving synergistic effects that exceed the sum of individual pathway modulation.

The integration of GLP-1 RAs into clinical practice requires systematic approaches that encompass evidence-based patient selection, structured initiation and monitoring protocols, multidisciplinary collaboration across medical specialties, and comprehensive care coordination to optimize therapeutic outcomes while ensuring patient safety. The expanding evidence base necessitates continuous provider education, clinical decision support tools, and adaptations to healthcare delivery models to accommodate the complexity of multi-indication prescribing and monitoring requirements.

Future research priorities encompass mechanistic investigations to enable next-generation therapeutic development, clinical trials exploring novel indications and combination approaches, technological innovations improving drug delivery and patient monitoring, and implementation research addressing real-world effectiveness and healthcare system integration. The success of continued GLP-1 RA development will require coordinated efforts across basic science, clinical research, regulatory science, and health services research to realize the full therapeutic potential while ensuring safe, equitable access for all patients who could benefit.

The evidence reviewed establishes GLP-1 RAs as transformative precision medicine tools that exemplify how mechanistic understanding can drive therapeutic innovation in addressing complex, interconnected diseases through targeted modulation of biological pathways. Their continued evolution promises further advances in an emerging medical paradigm where single interventions address multiple pathophysiological processes, improving outcomes while simplifying treatment complexity.

## Figures and Tables

**Table 1:** Search Strategy and Study Selection Criteria.

Search Databases	Inclusion Criteria	Exclusion Criteria
PubMed/MEDLINE	Original research articles	Conference abstracts only
Embase	Systematic reviews, meta-analyses	Case reports with <3 patients
Cochrane Library	Human clinical studies	Overlapping populations
Web of Science	Clear methodology	Inadequate methodology
ClinicalTrials.gov	English language	Language other than English

**Table 2:** Major GLP-1 Signaling Pathways and Their Effects.

Pathway	Mediators	Primary Effects	Clinical Relevance
cAMP/PKA	Gs, adenylyl cyclase, PKA, CREB	• Gene transcription• BDNF expression• Bcl-2 upregulation	Neuroprotection, cell survival
PI3K/Akt	PI3K, Akt, GSK-3β, mTORC1	• Cell survival• Protein synthesis• Tau phosphorylation inhibition	Metabolic regulation, neurodegeneration
β-Arrestin	β-arrestin-2, ERK	• Receptor desensitization• Sustained signaling• CREB phosphorylation	Receptor regulation, cellular survival
Wnt/β-catenin	GSK-3β inhibition, β-catenin	• Neurogenesis• β-cell proliferation• Tissue regeneration	Regenerative processes

**Table 3:** Mitochondrial Effects of GLP-1 Receptor Activation.

Effect	Mechanism	Functional Outcome
Biogenesis	PGC-1α upregulation	Increased mitochondrial number
Respiratory Capacity	Complex I/IV upregulation	Enhanced ATP production
Calcium Buffering	MCU regulation	Prevented Ca^2+^-induced dysfunction
Quality Control	PINK1/Parkin pathway	Damaged mitochondria removal
Dynamics	OPA1 upregulation	Maintained network integrity

**Table 4:** Comparative Efficacy of GLP-1 RAs in Type 2 Diabetes.

Agent	Dose	HbA1c Reduction (%)	Weight Loss (kg)	Key Trial	Reference
Tirzepatide	15 mg weekly	−2.01	−11.2	SURPASS-2	[9]
Semaglutide	1.0 mg weekly	−1.86	−6.9	SURPASS-2	[9]
Dulaglutide	1.5 mg weekly	−1.5	−3.1	REWIND	[59]
Liraglutide	1.8 mg daily	−1.3	−2.8	LEADER	[60]
Oral Semaglutide	14 mg daily	−1.2	−3.7	PIONEER 6	[61]

**Table 5:** Weight Loss Efficacy of GLP-1 RAs in Obesity.

Agent	Dose	Weight Loss (%)	Study Population	Duration	Reference
Retatrutide	12 mg weekly	−24.2	Obesity without diabetes	48 weeks	[72]
Tirzepatide	15 mg weekly	−22.5	Obesity without diabetes	72 weeks	[73]
Semaglutide	2.4 mg weekly	−14.9	Obesity without diabetes	68 weeks	[74]
Liraglutide	3.0 mg daily	−8.4	Obesity without diabetes	56 weeks	[75]

**Table 6:** Cardiovascular Outcomes with GLP-1 RAs.

Trial	Agent	Population	MACE Reduction	HR (95% CI)	Reference
SELECT	Semaglutide 2.4 mg	Obesity, no diabetes	20%	0.80 (0.72–0.90)	[82]
LEADER	Liraglutide 1.8 mg	T2DM, high CV risk	13%	0.87 (0.78–0.97)	[60]
SUSTAIN-6	Semaglutide 1.0 mg	T2DM, high CV risk	26%	0.74 (0.58–0.95)	[83]
REWIND	Dulaglutide 1.5 mg	T2DM, mixed risk	12%	0.88 (0.79–0.99)	
SURPASS-4	Tirzepatide (5, 10, 15 mg)	T2DM, high CV risk	26%	0.74 (0.51–1.08)	[84]
SURMOUNT-1		Obesity, no diabetes	Reduction in predicted risk of ASCVD	N/A	[73]
SOUL	Oral Semaglutide	T2DM, high CV risk	14%	0.86 (0.77–0.96)	[67]

**Table 7:** Heart Failure Outcomes with GLP-1 RAs.

Trial	Agent	Population	Primary Endpoint	Reduction	Reference
SUMMIT	Tirzepatide	HFpEF + obesity	CV death/HF events	38% (HR 0.62)	[90]
STEP-HFpEF	Semaglutide 2.4 mg	HFpEF + obesity	Symptoms/QoL	Significant improvement	[91]
STEP-HFpEF DM	Semaglutide 2.4 mg	HFpEF + obesity + T2DM	Symptoms/QoL	Significant improvement	[92]

**Table 8:** GLP-1 RAs in MASH Clinical Trials.

Trial	Agent	Dose	Primary Endpoint	Response Rate	Reference
ESSENCE	Semaglutide	2.4 mg weekly	MASH resolution	62.9% vs. 34.3% placebo	[100]
SYNERGY-NASH	Tirzepatide	15 mg weekly	MASH resolution	62% vs. 10% placebo	[101,102]
Survodutide Study	Survodutide	4.8 mg weekly	MASH improvement	83% vs. 18.2% placebo	[103]
LEAN	Liraglutide	1.8 mg daily	NASH resolution	39% vs. 9% placebo	[104]

**Table 10:** Dermatological Benefits of GLP-1 RAs.

Condition	Agent	Primary Outcome	Effect Size	Reference
Psoriasis	Various GLP-1 RAs	PASI score reduction	Significant improvement	[132,133,134]
Hidradenitis Suppurativa	GLP-1 RAs	Hurley stage improvement	*p* = 0.002	[135,136]
Wound Healing	Liraglutide	Healing acceleration	Demonstrated in T2DM	[137,138]

**Table 11:** Respiratory Benefits of GLP-1 RAs.

Condition	Effect	Risk Reduction	Population	Reference
General Respiratory Disease	Overall protection	14% (RR 0.86)	77,485 participants	[147,148]
COPD Exacerbations	Reduced exacerbations	18% (HR 0.82)	T2DM + COPD patients	[149]
Asthma Exacerbations	Fewer exacerbations	Significant reduction	T2DM + asthma patients	[150,151,152]
Pneumonia	Prevention	28% (HR 0.72)	COPD patients	[149]

**Table 12:** Sleep Disorder Applications.

Agent	Indication	Primary Outcome	Improvement	FDA Status	Reference
Tirzepatide	Moderate–severe OSA	AHI reduction	Up to 62.8%	Approved Dec 2024	[154,155,156]
		Clinical remission	42–50% vs. 14–16% placebo		
Semaglutide	OSA (investigational)	Sleep parameters	Under investigation	Investigational	[157,158,159,160]

**Table 13:** Safety Profile of GLP-1 RAs.

Category	Common AEs	Serious AEs	Contraindications	Monitoring Required
Gastrointestinal	• Nausea (20–40%)• Vomiting• Diarrhea	• Pancreatitis (rare)• Gastroparesis	• Gastroparesis• IBD (relative)	• Amylase/lipase• GI symptoms
Endocrine	• Injection site reactions	• Thyroid cancer risk• Hypoglycemia (with insulin)	• MTC history• MEN2 syndrome	• Thyroid monitoring• Glucose levels
Perioperative	• Delayed gastric emptying	• Aspiration risk	• Recent surgery	• NPO guidelines• Anesthesia consultation
Psychiatric	• Mood changes	• Suicidal ideation (rare)	• Active psychosis	• Mental health screening

**Table 14:** Research Priorities by Category.

Research Area	Priority Objectives	Timeline	Expected Impact
Mechanistic	• Tissue-specific signaling• Multi-receptor optimization• Personalized biomarkers	2–5 years	Enhanced therapeutic targeting
Clinical Development	• Neurological trials completion• Pediatric applications• Combination therapies	3–7 years	Expanded indications
Technology	• Long-acting formulations• Alternative delivery routes• Digital integration	2–5 years	Improved patient experience
Implementation	• Cost-effectiveness studies• Access improvement• Provider education	1–3 years	Population health impact

## Data Availability

No new data were created or analyzed in this study. Data sharing is not applicable to this article.

## References

[1] Westermeier F., Fisman E.Z. (2025). Glucagon-like peptide-1 receptor agonists (GLP-1RAs) and cardiometabolic protection: Historical development and future challenges. Cardiovasc. Diabetol..

[2] Theodorakis N., Nikolaou M. (2025). Integrated Management of Cardiovascular–Renal–Hepatic–Metabolic Syndrome: Expanding Roles of SGLT2is, GLP-1RAs, and GIP/GLP-1RAs. Biomedicines.

[3] Chun J.H., Butts A. (2020). Long-acting GLP-1RAs. J. Am. Acad. Physician Assist..

[4] Drucker D.J. (2024). Efficacy and Safety of GLP-1 Medicines for Type 2 Diabetes and Obesity. Diabetes Care.

[5] Holst J.J. (2019). The incretin system in healthy humans: The role of GIP and GLP-1. Metabolism.

[6] Andersen A., Lund A., Knop F.K., Vilsbøll T. (2018). Glucagon-like peptide 1 in health and disease. Nat. Rev. Endocrinol..

[7] Müller T.D., Blüher M., Tschöp M.H., DiMarchi R.D. (2022). Anti-obesity drug discovery: Advances and challenges. Nat. Rev. Drug Discov..

[8] Latif W., Lambrinos K.J., Patel P., Rodriguez R. (2025). Compare and Contrast the Glucagon-like Peptide-1 Receptor Agonists (GLP1RAs). https://www.ncbi.nlm.nih.gov/books/NBK572151.

[9] Frías J.P., Davies M.J., Rosenstock J., Pérez Manghi F.C., Fernández Landó L., Bergman B.K., Liu B., Cui X., Brown K. (2021). Tirzepatide versus Semaglutide Once Weekly in Patients with Type 2 Diabetes. N. Engl. J. Med..

[10] Pratley R.E., Aroda V.R., Lingvay I., Lüdemann J., Andreassen C., Navarria A., Viljoen A. (2018). Semaglutide versus dulaglutide once weekly in patients with type 2 diabetes (SUSTAIN 7): A randomised, open-label, phase 3b trial. Lancet Diabetes Endocrinol..

[11] Ansari S., Khoo B., Tan T. (2024). Targeting the incretin system in obesity and type 2 diabetes mellitus. Nat. Rev. Endocrinol..

[12] Liu S., Hu J., Zhao C., Liu H., He C. (2025). Comparative efficacy of incretin drugs on glycemic control, body weight, and blood pressure in adults with overweight or obesity and with/without type 2 diabetes: A systematic review and network meta-analysis. Front. Endocrinol..

[13] Madsbad S., Holst J.J. (2025). The promise of glucagon-like peptide 1 receptor agonists (GLP-1RA) for the treatment of obesity: A look at phase 2 and 3 pipelines. Expert Opin. Investig. Drugs.

[14] Dutta D., Nagendra L., Harish B., Sharma M., Joshi A., Hathur B., Kamrul-Hasan A.B.M. (2024). Efficacy and Safety of Cagrilintide Alone and in Combination with Semaglutide (Cagrisema) as Anti-Obesity Medications: A Systematic Review and Meta-Analysis. Indian J. Endocrinol. Metab..

[15] Liu Q.K. (2024). Mechanisms of action and therapeutic applications of GLP-1 and dual GIP/GLP-1 receptor agonists. Front. Endocrinol..

[16] Zheng Z., Zong Y., Ma Y., Tian Y., Pang Y., Zhang C., Gao J. (2024). Glucagon-like peptide-1 receptor: Mechanisms and advances in therapy. Signal Transduct. Target Ther..

[17] Siddeeque N., Hussein M.H., Abdelmaksoud A., Bishop J., Attia A.S., Elshazli R.M., Fawzy M.S., Toraih E.A. (2024). Neuroprotective effects of GLP-1 receptor agonists in neurodegenerative Disorders: A Large-Scale Propensity-Matched cohort study. Int. Immunopharmacol..

[18] Tipa R.O., Balan D.G., Georgescu M.T., Ignat L.A., Vacaroiu I.A., Georgescu D.E., Raducu L., Mihai D.A., Chiperi L., Balcangiu-Stroescu A. (2024). A Systematic Review of Semaglutide’s Influence on Cognitive Function in Preclinical Animal Models and Cell-Line Studies. Int. J. Mol. Sci..

[19] Zaccolo M., Kovanich D. (2025). Nanodomain c AMP signaling in cardiac pathophysiology: Potential for developing targeted therapeutic interventions. Physiol. Rev..

[20] Tomas A., Jones B., Leech C. (2020). New Insights into Beta-Cell GLP-1 Receptor and cAMP Signaling. J. Mol. Biol..

[21] Pan J., Yao Q., Wang Y., Chang S., Li C., Wu Y., Shen J., Yang R. (2024). The role of PI3K signaling pathway in Alzheimer’s disease. Front. Aging Neurosci..

[22] Reich N., Hölscher C. (2022). The neuroprotective effects of glucagon-like peptide 1 in Alzheimer’s and Parkinson’s disease: An in-depth review. Front. Neurosci..

[23] Zaïmia N., Obeid J., Varrault A., Sabatier J., Broca C., Gilon P., Costes S., Bertrand G., Ravier M.A. (2023). GLP-1 and GIP receptors signal through distinct β-arrestin 2-dependent pathways to regulate pancreatic β cell function. Cell Rep..

[24] McNeill S.M., Lu J., Marion C., Carino C., Inoue A., Zhao P., Sexton P.M., Wootten D. (2024). The role of G protein-coupled receptor kinases in GLP-1R β-arrestin recruitment and internalisation. Biochem. Pharmacol..

[25] Mayendraraj A., Rosenkilde M.M., Gasbjerg L.S. (2022). GLP-1 and GIP receptor signaling in beta cells—A review of receptor interactions and co-stimulation. Peptides.

[26] Liu L., Rashid M., Wess J. (2025). Regulation of GLP-1 and Glucagon Receptor Function by β-Arrestins in Metabolically Important Cell Types. Biochemistry.

[27] Au H.C.T., Zheng Y.J., Le G.H., Wong S., Teopiz K.M., Kwan A.T.H., Gill H., Badulescu S., Kyle V., Rosenblat J.D. (2025). Association of glucagon-like peptide-1 receptor agonists (GLP-1 RAs) and neurogenesis: A systematic review. Acta Neuropsychiatr..

[28] Zhao Y., Yu J., Ping F., Xu L., Li W., Zhang H., Li Y. (2022). Insulin and liraglutide attenuate brain pathology in diabetic mice by enhancing the Wnt/β-catenin signaling pathway. Exp. Ther. Med..

[29] Wei L., Gao J., Wang L., Tao Q., Tu C. (2023). Hippo/YAP signaling pathway: A new therapeutic target for diabetes mellitus and vascular complications. Ther. Adv. Endocrinol. Metab..

[30] Halling J.F., Pilegaard H. (2020). PGC-1α-mediated regulation of mitochondrial function and physiological implications. Appl. Physiol. Nutr. Metab..

[31] Liu W., Jalewa J., Sharma M., Li G., Li L., Hölscher C. (2015). Neuroprotective effects of lixisenatide and liraglutide in the 1-methyl-4-phenyl-1,2,3,6-tetrahydropyridine mouse model of Parkinson’s disease. Neuroscience.

[32] Wang C., Li Q., Wang W., Guo L., Guo C., Sun Y., Zhang J. (2015). GLP-1 contributes to increases in PGC-1α expression by downregulating miR-23a to reduce apoptosis. Biochem. Biophys. Res. Commun..

[33] Yip J.M.X., Chiang G.S.H., Lee I.C.J., Lehming-Teo R., Dai K., Dongol L., Wang L.Y., Teo D., Seah G.T., Lehming N. (2025). Mitochondria and the Repurposing of Diabetes Drugs for Off-Label Health Benefits. Int. J. Mol. Sci..

[34] Martinez de Morentin P.B., Gonzalez J.A., Dowsett G.K.C., Martynova Y., Yeo G.S.H., Sylantyev S., Heisler L.K. (2024). A brainstem to hypothalamic arcuate nucleus GABAergic circuit drives feeding. Curr. Biol..

[35] Hong C.T., Chen J.H., Hu C.J. (2024). Role of glucagon-like peptide-1 receptor agonists in Alzheimer’s disease and Parkinson’s disease. J. Biomed. Sci..

[36] Alharbi S.H. (2024). Anti-inflammatory role of glucagon-like peptide 1 receptor agonists and its clinical implications. Ther. Adv. Endocrinol. Metab..

[37] Lymperopoulos A., Borges J.I., Stoicovy R.A. (2024). Cyclic Adenosine Monophosphate in Cardiac and Sympathoadrenal GLP-1 Receptor Signaling: Focus on Anti-Inflammatory Effects. Pharmaceutics.

[38] Li X., Jiang X., Jiang M., Wang Z.F., Zhao T., Cao S.M., Li Q.M. (2023). GLP-1RAs inhibit the activation of the NLRP3 inflammasome signaling pathway to regulate mouse renal podocyte pyroptosis. Acta Diabetol..

[39] Satheesan A., Kumar J., Leela K.V., Murugesan R., Chaithanya V., Angelin M. (2024). Review on the role of nucleotide-binding oligomerization domain-like receptor protein 3 (NLRP3) inflammasome pathway in diabetes: Mechanistic insights and therapeutic implications. Inflammopharmacology.

[40] Zhang X., Yang X., Zhang S., Wang J., Wang M., Ma T., Wan M., Lv X., Yan T., Jia Y. (2023). Wei-Tong-Xin exerts anti-inflammatory effects through TLR4-mediated macrophages M1/M2 polarization and affects GLP-1 secretion. J. Pharm. Pharmacol..

[41] Youssef N., Noureldein M.H., Riachi M.E., Haddad A., Eid A.A. (2024). Macrophage polarization and signaling in diabetic kidney disease: A catalyst for disease progression. Am. J. Physiol.-Ren. Physiol..

[42] Sun H., Hao Y., Liu H., Gao F. (2025). The immunomodulatory effects of GLP-1 receptor agonists in neurogenerative diseases and ischemic stroke treatment. Front. Immunol..

[43] da Silva E.M., Yariwake V.Y., Alves R.W., de Araujo D.R., Andrade-Oliveira V. (2022). Crosstalk between incretin hormones, Th17 and Treg cells in inflammatory diseases. Peptides.

[44] Schooling C.M., Yang G., Soliman G.A., Leung G.M. (2025). A Hypothesis That Glucagon-like Peptide-1 Receptor Agonists Exert Immediate and Multifaceted Effects by Activating Adenosine Monophosphate-Activate Protein Kinase (AMPK). Life.

[45] Wu Y., Wang H., Xu H. (2025). Autophagy-lysosome pathway in insulin & glucagon homeostasis. Front. Endocrinol..

[46] Lu G., Wang Y., Shi Y., Zhang Z., Huang C., He W., Wang C., Shen H.M. (2022). Autophagy in health and disease: From molecular mechanisms to therapeutic target. MedComm.

[47] Wang N., Zhou Y., Erasto Ngowi E., Qiao A. (2024). Autophagy: Playing an important role in diabetes and its complications. Med. Drug Discov..

[48] Fang Y., Ji L., Zhu C., Xiao Y., Zhang J., Lu J., Yin J., Wei L. (2020). Liraglutide Alleviates Hepatic Steatosis by Activating the TFEB-Regulated Autophagy-Lysosomal Pathway. Front. Cell Dev. Biol..

[49] Apostolova N., Vezza T., Muntane J., Rocha M., Víctor V.M. (2023). Mitochondrial Dysfunction and Mitophagy in Type 2 Diabetes: Pathophysiology and Therapeutic Targets. Antioxid. Redox. Signal.

[50] Yasasilka X.R., Lee M. (2024). Role of β-cell autophagy in β-cell physiology and the development of diabetes. J. Diabetes Investig..

[51] Nauck M.A., Meier J.J. (2016). The incretin effect in healthy individuals and those with type 2 diabetes: Physiology, pathophysiology, and response to therapeutic interventions. Lancet Diabetes Endocrinol..

[52] Wan W., Qin Q., Xie L., Zhang H., Wu F., Stevens R.C., Liu Y. (2023). GLP-1R Signaling and Functional Molecules in Incretin Therapy. Molecules.

[53] Campbell J.E., Newgard C.B. (2021). Mechanisms controlling pancreatic islet cell function in insulin secretion. Nat. Rev. Mol. Cell Biol..

[54] Baggio L.L., Drucker D.J. (2021). Glucagon-like peptide-1 receptor co-agonists for treating metabolic disease. Mol. Metab..

[55] Rowlands J., Heng J., Newsholme P., Carlessi R. (2018). Pleiotropic Effects of GLP-1 and Analogs on Cell Signaling, Metabolism, and Function. Front. Endocrinol..

[56] Gribble F.M., Reimann F. (2019). Function and mechanisms of enteroendocrine cells and gut hormones in metabolism. Nat. Rev. Endocrinol..

[57] Kaneto H., Kimura T., Shimoda M., Obata A., Sanada J., Fushimi Y., Nakanishi S., Mune T., Kaku K. (2021). Favorable Effects of GLP-1 Receptor Agonist against Pancreatic β-Cell Glucose Toxicity and the Development of Arteriosclerosis: “The Earlier, the Better” in Therapy with Incretin-Based Medicine. Int. J. Mol. Sci..

[58] Hinnen D. (2017). Glucagon-Like Peptide 1 Receptor Agonists for Type 2 Diabetes. Diabetes Spectr..

[59] Gerstein H.C., Colhoun H.M., Dagenais G.R., Diaz R., Lakshmanan M., Pais P., Probstfield J., Riesmeyer J.S., Riddle M.C., Rydén L. (2019). Dulaglutide and cardiovascular outcomes in type 2 diabetes (REWIND): A double-blind, randomised placebo-controlled trial. Lancet.

[60] Marso S.P., Daniels G.H., Brown-Frandsen K., Kristensen P., Mann J.F.E., Nauck M.A., Nissen S.E., Pocock S., Poulter N.R., Ravn L.S. (2016). Liraglutide and Cardiovascular Outcomes in Type 2 Diabetes. N. Engl. J. Med..

[61] Husain M., Birkenfeld A.L., Donsmark M., Dungan K., Eliaschewitz F.G., Franco D.R., Jeppesen O.K., Lingvay I., Mosenzon O., Pedersen S.D. (2019). Oral Semaglutide and Cardiovascular Outcomes in Patients with Type 2 Diabetes. N. Engl. J. Med..

[62] Yao H., Zhang A., Li D., Wu Y., Wang C.Z., Wan J.Y., Yuan C.S. (2024). Comparative effectiveness of GLP-1 receptor agonists on glycaemic control, body weight, and lipid profile for type 2 diabetes: Systematic review and network meta-analysis. BMJ.

[63] Terrell K., Vallarino C.R., Lozada J.M., Grabner M., Teng C.C., Hoog M.M., Richard E. (2025). Real-World Effectiveness of Tirzepatide vs. Semaglutide on HbA1c and Weight in GLP-1 RA Naïve Patients with T2D. In: ISPOR [Internet]. https://www.ispor.org/heor-resources/presentations-database/presentation-cti/ispor-2025/poster-session-1/real-world-effectiveness-of-tirzepatide-vs-semaglutide-on-hba1c-and-weight-in-glp-1-ra-na-239-ve-patients-with-t2d.

[64] Lv L., Wang Y., Xie L., Noone J., Alvarez S., Zhang Y., Song Y., Rotroff D.M. (2025). Newer Glucagon-Like Peptide-1 Receptor Agonists Are Associated with Improved Glycemic Control in US Adults with Type 2 Diabetes: A Population-Level Time Series Analysis. Value Health.

[65] Marassi M., Fadini G.P. (2025). Real-world Evidence on Oral Semaglutide for the Management of Type 2 Diabetes. A Narrative Review for Clinical Practice. Clin. Ther..

[66] Lee M.M.Y., Sattar N., Pop-Busui R., Deanfield J., Emerson S.S., Inzucchi S.E., Mann J.F.E., Marx N., Mulvagh S.L., Poulter N.R. (2025). Cardiovascular and Kidney Outcomes and Mortality with Long-Acting Injectable and Oral Glucagon-Like Peptide 1 Receptor Agonists in Individuals with Type 2 Diabetes: A Systematic Review and Meta-analysis of Randomized Trials. Diabetes Care.

[67] McGuire D.K., Marx N., Mulvagh S.L., Deanfield J.E., Inzucchi S.E., Pop-Busui R., Mann J.F.E., Emerson S.S., Poulter N.R., Engelmann M.D.M. (2025). Oral Semaglutide and Cardiovascular Outcomes in High-Risk Type 2 Diabetes. N. Engl. J. Med..

[68] Dimitrios P., Michael D., Vasilios K., Konstantinos S., Konstantinos I., Ioanna Z., Konstantinos P., Spyridon B., Asterios K. (2020). Liraglutide as Adjunct to Insulin Treatment in Patients with Type 1 Diabetes: A Systematic Review and Meta-analysis. Curr. Diabetes Rev..

[69] Park J., Ntelis S., Yunasan E., Downton K.D., Yip T.C.F., Munir K.M., Haq N. (2023). Glucagon-Like Peptide 1 Analogues as Adjunctive Therapy for Patients with Type 1 Diabetes: An Updated Systematic Review and Meta-analysis. J. Clin. Endocrinol. Metab..

[70] Brierley D.I., de Lartigue G. (2022). Reappraising the role of the vagus nerve in GLP-1-mediated regulation of eating. Br. J. Pharmacol..

[71] Dong Y., Carty J., Goldstein N., He Z., Hwang E., Chau D., Wallace B., Kabahizi A., Lieu L., Peng Y. (2021). Time and metabolic state-dependent effects of GLP-1R agonists on NPY/AgRP and POMC neuronal activity in vivo. Mol. Metab..

[72] Jastreboff A.M., Kaplan L.M., Frías J.P., Wu Q., Du Y., Gurbuz S., Coskun T., Haupt A., Milicevic Z., Hartman M.L. (2023). Triple–Hormone-Receptor Agonist Retatrutide for Obesity—A Phase 2 Trial. N. Engl. J. Med..

[73] Jastreboff A.M., Aronne L.J., Ahmad N.N., Wharton S., Connery L., Alves B., Kiyosue A., Zhang S., Liu B., Bunck M.C. (2022). Tirzepatide Once Weekly for the Treatment of Obesity. N. Engl. J. Med..

[74] Wilding J.P.H., Batterham R.L., Calanna S., Davies M., Van Gaal L.F., Lingvay I., McGowan B.M., Rosenstock J., Tran M.T.D., Wadden T.A. (2021). Once-Weekly Semaglutide in Adults with Overweight or Obesity. N. Engl. J. Med..

[75] Pi-Sunyer X., Astrup A., Fujioka K., Greenway F., Halpern A., Krempf M., Lau D.C.W., Roux C.W., Ortiz R.V., Jensen C.B. (2015). A Randomized, Controlled Trial of 3.0 mg of Liraglutide in Weight Management. N. Engl. J. Med..

[76] Liu Y., Ruan B., Jiang H., Le S., Liu Y., Ao X., Huang Y., Shi X., Xue R., Fu X. (2023). The Weight-loss Effect of GLP-1RAs Glucagon-Like Peptide-1 Receptor Agonists in Non-diabetic Individuals with Overweight or Obesity: A Systematic Review with Meta-Analysis and Trial Sequential Analysis of Randomized Controlled Trials. Am. J. Clin. Nutr..

[77] Guo H., Yang J., Huang J., Xu L., Lv Y., Wang Y., Ren J., Feng Y., Zheng Q., Li L. (2025). Comparative efficacy and safety of GLP-1 receptor agonists for weight reduction: A model-based meta-analysis of placebo-controlled trials. Obes. Pillars.

[78] Levy M.E., Schiabor Barrett K.M., Cirulli E.T. (2025). Semaglutide vs Tirzepatide Dosages for Weight Loss. JAMA Intern. Med..

[79] Abdrabou Abouelmagd A., Abdelrehim A.M., Bashir M.N., Abdelsalam F., Marey A., Tanas Y., Abuklish D.M., Belal M.M. (2025). Efficacy and safety of retatrutide, a novel GLP-1, GIP, and glucagon receptor agonist for obesity treatment: A systematic review and meta-analysis of randomized controlled trials. Bayl. Univ. Med. Cent. Proc..

[80] Tewari J., Qidwai K.A., Tewari A., Kaur S., Tewari V., Maheshwari A. (2025). Efficacy and safety of triple hormone receptor agonist retatrutide for the management of obesity: A systematic review and meta-analysis. Expert Rev. Clin. Pharmacol..

[81] Le R., Nguyen M.T., Allahwala M.A., Psaltis J.P., Marathe C.S., Marathe J.A., Psaltis P.J. (2024). Cardiovascular Protective Properties of GLP-1 Receptor Agonists: More than Just Diabetic and Weight Loss Drugs. J. Clin. Med..

[82] Lincoff A.M., Brown-Frandsen K., Colhoun H.M., Deanfield J., Emerson S.S., Esbjerg S., Hardt-Lindberg S., Hovingh G.K., Kahn S.E., Kushner R.F. (2023). Semaglutide and Cardiovascular Outcomes in Obesity without Diabetes. N. Engl. J. Med..

[83] Marso S.P., Bain S.C., Consoli A., Eliaschewitz F.G., Jódar E., Leiter L.A., Lingvay I., Rosenstock J., Seufert J., Warren M.L. (2016). Semaglutide and Cardiovascular Outcomes in Patients with Type 2 Diabetes. N. Engl. J. Med..

[84] Del Prato S., Kahn S.E., Pavo I., Weerakkody G.J., Yang Z., Doupis J., Aizenberg D., Wynne A.G., Riesmeyer J.S., Heine R.J. (2021). Tirzepatide versus insulin glargine in type 2 diabetes and increased cardiovascular risk (SURPASS-4): A randomised, open-label, parallel-group, multicentre, phase 3 trial. Lancet.

[85] Giugliano D., Scappaticcio L., Longo M., Caruso P., Maiorino M.I., Bellastella G., Ceriello A., Chiodini P., Esposito K. (2021). GLP-1 receptor agonists and cardiorenal outcomes in type 2 diabetes: An updated meta-analysis of eight CVOTs. Cardiovasc. Diabetol..

[86] Pfeffer M.A., Claggett B., Diaz R., Dickstein K., Gerstein H.C., Køber L.V., Lawson F.C., Ping L., Wei X., Lewis E.F. (2015). Lixisenatide in Patients with Type 2 Diabetes and Acute Coronary Syndrome. N. Engl. J. Med..

[87] Rivera F.B., Cruz L.L.A., Magalong J.V., Ruyeras J.M.M.J., Aparece J.P., Bantayan N.R.B., Lara-Breitinger K., Gulati M. (2024). Cardiovascular and renal outcomes of glucagon-like peptide 1 receptor agonists among patients with and without type 2 diabetes mellitus: A meta-analysis of randomized placebo-controlled trials. Am. J. Prev. Cardiol..

[88] Nicholls S.J., Bhatt D.L., Buse J.B., Del Prato S., Kahn S.E., Lincoff A.M., McGuire D.K., Nauck M.A., Nissen S.E., Sattar N. (2024). Comparison of tirzepatide and dulaglutide on major adverse cardiovascular events in participants with type 2 diabetes and atherosclerotic cardiovascular disease: SURPASS-CVOT design and baseline characteristics. Am. Heart J..

[89] Schnell O., Almandoz J., Anderson L., Barnard-Kelly K., Battelino T., Blüher M., Busetto L., Catrinou D., Ceriello A., Cos X. (2025). CVOT summit report 2024: New cardiovascular, kidney, and metabolic outcomes. Cardiovasc. Diabetol..

[90] Packer M., Zile M.R., Kramer C.M., Baum S.J., Litwin S.E., Menon V., Ge J., Weerakkody G.J., Ou Y., Bunck M.C. (2025). Tirzepatide for Heart Failure with Preserved Ejection Fraction and Obesity. N. Engl. J. Med..

[91] Kosiborod M.N., Abildstrøm S.Z., Borlaug B.A., Butler J., Rasmussen S., Davies M., Hovingh G.K., Kitzman D.W., Lindegaard M.L., Møller D.V. (2023). Semaglutide in Patients with Heart Failure with Preserved Ejection Fraction and Obesity. N. Engl. J. Med..

[92] Kosiborod M.N., Petrie M.C., Borlaug B.A., Butler J., Davies M.J., Hovingh G.K., Kitzman D.W., Møller D.V., Treppendahl M.B., Verma S. (2024). Semaglutide in Patients with Obesity-Related Heart Failure and Type 2 Diabetes. N. Engl. J. Med..

[93] Kosiborod M.N., Deanfield J., Pratley R., Borlaug B.A., Butler J., Davies M.J., Emerson S.S., Kahn S.E., Kitzman D.W., Lingvay I. (2024). Semaglutide versus placebo in patients with heart failure and mildly reduced or preserved ejection fraction: A pooled analysis of the SELECT, FLOW, STEP-HFpEF, and STEP-HFpEF DM randomised trials. Lancet.

[94] Badve S.V., Bilal A., Lee M.M.Y., Sattar N., Gerstein H.C., Ruff C.T., McMurray J.J.V., Rossing P., Bakris G., Mahaffey K.W. (2025). Effects of GLP-1 receptor agonists on kidney and cardiovascular disease outcomes: A meta-analysis of randomised controlled trials. Lancet Diabetes Endocrinol..

[95] Parvanova A., Abbate M., Reseghetti E., Ruggenenti P. (2025). Mechanisms and treatment of obesity-related hypertension—Part 2: Treatments. Clin Kidney J..

[96] Morales E., Martin W.P., Bevc S., Jenssen T.G., Miglinas M., Trillini M. (2025). How to individualise renoprotective therapy in obese patients with chronic kidney disease: A commentary by the Diabesity Working Group of the ERA. Nephrol. Dial. Transplant..

[97] Trevella P., Ekinci E.I., MacIsaac R.J. (2024). Potential kidney protective effects of glucagon-like peptide-1 receptor agonists. Nephrology.

[98] Younossi Z.M., Kalligeros M., Henry L. (2025). Epidemiology of metabolic dysfunction-associated steatotic liver disease. Clin. Mol. Hepatol..

[99] Abushamat L.A., Shah P.A., Eckel R.H., Harrison S.A., Barb D. (2024). The Emerging Role of Glucagon-Like Peptide-1 Receptor Agonists for the Treatment of Metabolic Dysfunction-Associated Steatohepatitis. Clin. Gastroenterol. Hepatol..

[100] Sanyal A.J., Newsome P.N., Kliers I., Østergaard L.H., Long M.T., Kjær M.S., Cali A.M.G., Bugianesi E., Rinella M.E., Roden M. (2025). Phase 3 Trial of Semaglutide in Metabolic Dysfunction–Associated Steatohepatitis. N. Engl. J. Med..

[101] Vuppalanchi R., Loomba R., Sanyal A.J., Nikooie A., Tang Y., Robins D.A., Brouwers B., Hartman M.L. (2024). Randomised clinical trial: Design of the SYNERGY-NASH phase 2b trial to evaluate tirzepatide as a treatment for metabolic dysfunction-associated steatohepatitis and modification of screening strategy to reduce screen failures. Aliment Pharmacol. Ther..

[102] Loomba R., Hartman M.L., Lawitz E.J., Vuppalanchi R., Boursier J., Bugianesi E., Yoneda M., Behling C., Cummings O.W., Tang Y. (2024). Tirzepatide for Metabolic Dysfunction–Associated Steatohepatitis with Liver Fibrosis. N. Engl. J. Med..

[103] Sanyal A.J., Bedossa P., Fraessdorf M., Neff G.W., Lawitz E., Bugianesi E., Anstee Q.M., Hussain S.A., Newsome P.N., Ratziu V. (2024). A Phase 2 Randomized Trial of Survodutide in MASH and Fibrosis. N. Engl. J. Med..

[104] Armstrong M.J., Gaunt P., Aithal G.P., Barton D., Hull D., Parker R., Hazlehurst J.M., Guo K., Abouda G., Aldersley M.A. (2016). Liraglutide safety and efficacy in patients with non-alcoholic steatohepatitis (LEAN): A multicentre, double-blind, randomised, placebo-controlled phase 2 study. Lancet.

[105] An H., Jang Y., Choi J., Hur J., Kim S., Kwon Y. (2025). New Insights into AMPK, as a Potential Therapeutic Target in Metabolic Dysfunction-Associated Steatotic Liver Disease and Hepatic Fibrosis. Biomol. Ther..

[106] De Cól J.P., de Lima E.P., Pompeu F.M., Cressoni Araújo A., de Alvares Goulart R., Bechara M.D., Laurindo L.F., Mén-dez-Sánchez N., Barbalho S.M. (2024). Underlying Mechanisms behind the Brain–Gut–Liver Axis and Metabolic-Associated Fatty Liver Disease (MAFLD): An Update. Int. J. Mol. Sci..

[107] Cazac-Panaite G.D., Lăcătușu C.M., Grigorescu E.D., Foșălău A.B., Onofriescu A., Mihai B.M. (2025). Innovative Drugs First Implemented in Type 2 Diabetes Mellitus and Obesity and Their Effects on Metabolic Dysfunction-Associated Steatohepatitis (MASH)-Related Fibrosis and Cirrhosis. J. Clin. Med..

[108] Targher G., Mantovani A., Byrne C.D., Tilg H. (2025). Recent advances in incretin-based therapy for MASLD: From single to dual or triple incretin receptor agonists. Gut.

[109] Wang M., Yoon G., Song J., Jo J. (2021). Exendin-4 improves long-term potentiation and neuronal dendritic growth in vivo and in vitro obesity condition. Sci. Rep..

[110] Femminella G.D., Frangou E., Love S.B., Busza G., Holmes C., Ritchie C., Lawrence R., McFarlane B., Tadros G., Ridha B.H. (2019). Evaluating the effects of the novel GLP-1 analogue liraglutide in Alzheimer’s disease: Study protocol for a randomised controlled trial (ELAD study). Trials.

[111] Scheltens P., Atri A., Feldman H., Hansson O., Knop F., Sano M., Dethlefsen C., Johannsen P., León T., Hansen C.T. (2024). Baseline Characteristics from Evoke and Evoke+: Two Phase 3 Randomized Placebo-controlled Trials of Oral Semaglutide in Patients with Early Alzheimer’s Disease (P11-9.013). Neurology.

[112] Cummings J.L., Atri A., Feldman H.H., Hansson O., Sano M., Knop F.K., Johannsen P., León T., Scheltens P. (2025). evoke and evoke+: Design of two large-scale, double-blind, placebo-controlled, phase 3 studies evaluating efficacy, safety, and tolerability of semaglutide in early-stage symptomatic Alzheimer’s disease. Alzheimers Res. Ther..

[113] Mekhail N.A., Levy R.M., Deer T.R., Kapural L., Li S., Amirdelfan K., Pope J.E., Hunter C.W., Rosen S.M., Costandi S.J. (2024). ECAP-controlled closed-loop versus open-loop SCS for the treatment of chronic pain: 36-month results of the EVOKE blinded randomized clinical trial. Reg. Anesth. Pain Med..

[114] Vijiaratnam N., Girges C., Auld G., Chau M., Maclagan K., King A., Skene S., Chowdhury K., Hibbert S., Morris H. (2021). Exenatide once weekly over 2 years as a potential disease-modifying treatment for Parkinson’s disease: Protocol for a multicentre, randomised, double blind, parallel group, placebo controlled, phase 3 trial: The ‘Exenatide-PD3’ study. BMJ Open.

[115] Meissner W.G., Remy P., Giordana C., Maltête D., Derkinderen P., Houéto J.L., Anheim M., Benatru I., Boraud T., Bre-fel-Courbon C. (2024). Trial of Lixisenatide in Early Parkinson’s Disease. N. Engl. J. Med..

[116] Mahapatra M.K., Karuppasamy M., Sahoo B.M. (2022). Therapeutic Potential of Semaglutide, a Newer GLP-1 Receptor Agonist, in Abating Obesity, Non-Alcoholic Steatohepatitis and Neurodegenerative diseases: A Narrative Review. Pharm. Res..

[117] McFarthing K., Larson D., Simuni T. (2020). Clinical Trial Highlights—GLP-1 agonists. J. Park. Dis..

[118] Teixeira L.C.R., Luizon M.R., Gomes K.B. (2025). Exploring the Role of GLP-1 Receptor Agonists in Alzheimer’s Disease: A Review of Preclinical and Clinical Evidence. Receptors.

[119] Urkon M., Ferencz E., Szász J.A., Szabo M.I.M., Orbán-Kis K., Szatmári S., Nagy E.E. (2025). Antidiabetic GLP-1 Receptor Agonists Have Neuroprotective Properties in Experimental Animal Models of Alzheimer’s Disease. Pharmaceuticals.

[120] Duță C., Muscurel C., Dogaru C.B., Stoian I. (2025). Targeting Ferroptosis in Parkinson’s: Repurposing Diabetes Drugs as a Promising Treatment. Int. J. Mol. Sci..

[121] Vijiaratnam N., Girges C., Auld G., McComish R., King A., Skene S.S., Hibbert S., Wong A., Melander S., Gibson R. (2025). Exenatide once a week versus placebo as a potential disease-modifying treatment for people with Parkinson’s disease in the UK: A phase 3, multicentre, double-blind, parallel-group, randomised, placebo-controlled trial. Lancet.

[122] Xie Y., Choi T., Al-Aly Z. (2025). Mapping the effectiveness and risks of GLP-1 receptor agonists. Nat. Med..

[123] Sánchez-Garrido M.A., Serrano-López V., Ruiz-Pino F., Vázquez M.J., Rodríguez-Martín A., Torres E., Velasco I., Rodríguez A.B., Chicano-Gálvez E., Mora-Ortiz M. (2024). Superior metabolic improvement of polycystic ovary syndrome traits after GLP1-based multi-agonist therapy. Nat. Commun..

[124] Houston E.J., Templeman N.M. (2025). Reappraising the relationship between hyperinsulinemia and insulin resistance in PCOS. J. Endocrinol..

[125] Dong J., Rees D.A. (2023). Polycystic ovary syndrome: Pathophysiology and therapeutic opportunities. BMJ Med..

[126] Rashid R., Mir S.A., Kareem O., Ali T., Ara R., Malik A., Amin F., Bader G.N. (2022). Polycystic ovarian syndrome-current pharmacotherapy and clinical implications. Taiwan J. Obs. Gynecol..

[127] Monney M., Mavromati M., Leboulleux S., Gariani K. (2025). Endocrine and metabolic effects of GLP-1 receptor agonists on women with PCOS, a narrative review. Endocr. Connect..

[128] Han Y., Li Y., He B. (2019). GLP-1 receptor agonists versus metformin in PCOS: A systematic review and meta-analysis. Reprod. Biomed. Online.

[129] Austregésilo de Athayde De Hollanda Morais B., Martins Prizão V., de Moura de Souza M., Ximenes Mendes B., Rodrigues Defante M.L., Cosendey Martins O., Rodrigues A.M. (2024). The efficacy and safety of GLP-1 agonists in PCOS women living with obesity in promoting weight loss and hormonal regulation: A meta-analysis of randomized controlled trials. J. Diabetes Complicat..

[130] Zhou L., Qu H., Yang L., Shou L. (2023). Effects of GLP1RAs on pregnancy rate and menstrual cyclicity in women with polycystic ovary syndrome: A meta-analysis and systematic review. BMC Endocr. Disord..

[131] Patino W., Thomas A., Jain S., Del Rosso J.Q., Issa N.T. (2025). A Review of Glucagon-like Peptide-1 in Dermatology. J. Clin. Aesthet. Dermatol..

[132] Costanzo G., Curatolo S., Busà B., Belfiore A., Gullo D. (2021). Two birds one stone: Semaglutide is highly effective against severe psoriasis in a type 2 diabetic patient. Endocrinol. Diabetes Metab. Case Rep..

[133] Ku S., Chang H. (2024). Efficacy of glucagon-like peptide-1 receptor agonists for psoriasis: An updated systematic review and meta-analysis. JDDG J. Der Dtsch. Dermatol. Ges..

[134] Haran K., Johnson C.E., Smith P., Venable Z., Kranyak A., Bhutani T., Liao W. (2024). Impact of GLP-1 Receptor Agonists on Psoriasis and Cardiovascular Comorbidities: A Narrative Review. Psoriasis Targets Ther..

[135] Taylor S. (2025). GLP-1 RAs and Hidradenitis Suppurativa in Patients with Overweight or Obesity, with Susan Taylor, MD. In: American Academy of Dermatology [Internet]. https://www.hcplive.com/view/glp-1-ras-and-hidradenitis-suppurativa-in-patients-with-overweight-or-obesity-with-susan-taylor-md.

[136] Krajewski P.K., Złotowska A., Szepietowski J.C. (2024). The Therapeutic Potential of GLP-1 Receptor Agonists in the Management of Hidradenitis Suppurativa: A Systematic Review of Anti-Inflammatory and Metabolic Effects. J. Clin. Med..

[137] Zhang Q., Zhang C., Kang C., Zhu J., He Q., Li H., Xu H., Liu Y., Wang L., Chen S. (2024). Liraglutide Promotes Diabetic Wound Healing via Myo1c/Dock5. Adv. Sci..

[138] Dhatariya K., Bain S.C., Buse J.B., Simpson R., Tarnow L., Kaltoft M.S., Stellfeld M., Tornøe K., Pratley R.E., LEADER Publication Committee on behalf of the LEADER Trial Investigators (2018). The Impact of Liraglutide on Diabetes-Related Foot Ulceration and Associated Complications in Patients with Type 2 Diabetes at High Risk for Cardiovascular Events: Results from the LEADER Trial. Diabetes Care.

[139] Yang J., Wang Z., Zhang X. (2019). GLP-1 receptor agonist impairs keratinocytes inflammatory signals by activating AMPK. Exp. Mol. Pathol..

[140] Burke O.M., Sa B., Cespedes D.A., Tosti A. (2025). Dermatologic Implications of GLP-1 Receptor Agonist Medications: A Comprehensive Review. Skin Appendage Disord..

[141] Persson C., Eaton A., Mayrovitz H.N. (2025). A Closer Look at the Dermatological Profile of GLP-1 Agonists. Diseases.

[142] Heymann W.R. (2024). Weighing in on glucagon-like peptide-1 receptor agonists in dermatology. J. Am. Acad. Dermatol..

[143] Karacabeyli D., Lacaille D. (2024). Glucagon-Like Peptide 1 Receptor Agonists in Patients with Inflammatory Arthritis or Psoriasis. JCR J. Clin. Rheumatol..

[144] Petković-Dabić J., Binić I., Carić B., Božić L., Umičević-Šipka S., Bednarčuk N., Dabić S., Šitum M., Popović-Pejičić S., Stojiljković M.P. (2025). Effects of Semaglutide Treatment on Psoriatic Lesions in Obese Patients with Type 2 Diabetes Mellitus: An Open-Label, Randomized Clinical Trial. Biomolecules.

[145] Janić M., Škrgat S., Harlander M., Lunder M., Janež A., Pantea Stoian A., El-Tanani M., Maggio V., Rizzo M. (2024). Potential Use of GLP-1 and GIP/GLP-1 Receptor Agonists for Respiratory Disorders: Where Are We at?. Medicina (B Aires).

[146] Wang W., Mei A., Qian H., Li D., Xu H., Chen J., Yang H., Min X., Li C., Cheng L. (2023). The Role of Glucagon-Like Peptide-1 Receptor Agonists in Chronic Obstructive Pulmonary Disease. Int. J. Chronic Obstr. Pulm. Dis..

[147] Yu M., Wang R., Pei L., Zhang X., Wei J., Wen Y., Liu H., Ye H., Wang J., Wang L. (2023). The relationship between the use of GLP-1 receptor agonists and the incidence of respiratory illness: A meta-analysis of randomized controlled trials. Diabetol. Metab. Syndr..

[148] Cooper D.H., Akbarian N., Aaron S.D., Luks V., Kendzerska T. (2025). Glucagon-like peptide 1 (GLP-1) receptor agonists and asthma and COPD exacerbations in adults with diabetes: A systematic review. Respir. Med..

[149] See X.Y., Xanthavanij N., Lee Y.C., Ong T.E., Wang T.H., Ahmed O., Chang Y.C., Peng C.Y., Chi K.Y., Chang Y. (2025). Pulmonary outcomes of incretin-based therapies in COPD patients receiving single-inhaler triple therapy. ERJ Open Res..

[150] Foer D., Beeler P.E., Cui J., Karlson E.W., Bates D.W., Cahill K.N. (2021). Asthma Exacerbations in Patients with Type 2 Diabetes and Asthma on Glucagon-like Peptide-1 Receptor Agonists. Am. J. Respir. Crit. Care Med..

[151] Wang T., Keil A.P., Buse J.B., Keet C., Kim S., Wyss R., Pate V., Jonsson-Funk M., Pratley R.E., Kvist K. (2024). Glucagon-like Peptide 1 Receptor Agonists and Asthma Exacerbations: Which Patients Benefit Most?. Ann. Am. Thorac. Soc..

[152] Zhang M.Q., Lin C., Cai X.L., Jiao R.Y., Bai S.Z., Li Z.L., Hu S.Y., Lyu F., Yang W.J., Ji L.N. (2024). The Association between GLP-1 Receptor-Based Agonists and the Incidence of Asthma in Patients with Type 2 Diabetes and/or Obesity: A Meta-Analysis. Biomed. Environ. Sci..

[153] Kantreva K., Katsaounou P., Saltiki K., Trakada G., Ntali G., Stratigou T., Tzanela M., Psaltopoulou T., Paschou S.A. (2024). The possible effect of anti-diabetic agents GLP-1RA and SGLT-2i on the respiratory system function. Endocrine.

[154] (2024). USFDA FDA News Release. FDA Approves First Medication for Obstructive Sleep Apnea. https://www.fda.gov/news-events/press-announcements/.

[155] Malhotra A., Grunstein R.R., Fietze I., Weaver T.E., Redline S., Azarbarzin A., Sands S.A., Schwab R.J., Dunn J.P., Chakladar S. (2024). Tirzepatide for the Treatment of Obstructive Sleep Apnea and Obesity. N. Engl. J. Med..

[156] Malhotra A., Bednarik J., Chakladar S., Dunn J.P., Weaver T., Grunstein R., Fietze I., Redline S., Azarbarzin A., Sands S.A. (2024). Tirzepatide for the treatment of obstructive sleep apnea: Rationale, design, and sample baseline characteristics of the SURMOUNT -OSA phase 3 trial. Contemp. Clin. Trials.

[157] Baser O., Lu Y., Chen S., Chen S., Baser E. (2024). Tirzepatide and Semaglutide for the Treatment of Obstructive Sleep Apnea and Obesity: A Retrospective Analysis. Med. Res. Arch..

[158] Li M., Lin H., Yang Q., Zhang X., Zhou Q., Shi J., Ge F. (2025). Glucagon-like peptide-1 receptor agonists for the treatment of obstructive sleep apnea: A meta-analysis. Sleep.

[159] Yang R., Zhang L., Guo J., Wang N., Zhang Q., Qi Z., Wu L., Qin L., Liu T. (2025). Glucagon-like Peptide-1 receptor agonists for obstructive sleep apnea in patients with obesity and type 2 diabetes mellitus: A systematic review and meta-analysis. J. Transl. Med..

[160] Le K.D.R., Le K., Foo F. (2024). The Impact of Glucagon-like Peptide 1 Receptor Agonists on Obstructive Sleep Apnoea: A Scoping Review. Pharmacy.

[161] Beccuti G., Bioletto F., Parasiliti-Caprino M., Benso A., Ghigo E., Cicolin A., Broglio F. (2024). Estimating Cardiovascular Benefits of Tirzepatide in Sleep Apnea and Obesity: Insight from the SURMOUNT-OSA Trials. Curr. Obes. Rep..

[162] El-Solh A.A., Gould E., Aibangbee K., Jimerson T., Hartling R. (2025). Current perspectives on the use of GLP-1 receptor agonists in obesity-related obstructive sleep apnea: A narrative review. Expert Opin. Pharmacother..

[163] Romariz L., Araújo B., Barbosa L.M., Jain R., Porto Silva Janovsky C.C. (2025). GLP-1 receptor agonists for the treatment of obstructive sleep apnea and obesity. Eur. J. Intern. Med..

[164] Dragonieri S., Portacci A., Quaranta V.N., Carratu P., Lazar Z., Carpagnano G.E., Resta O., Portincasa P., Palmieri V.O. (2024). Therapeutic Potential of Glucagon-like Peptide-1 Receptor Agonists in Obstructive Sleep Apnea Syndrome Management: A Narrative Review. Diseases.

[165] Huang L., Feng T. (2025). The Application of GLP-1 Receptor Agonists and SGLT2 Inhibitors in Obstructive Sleep Apnea: Breakthrough or Overhyped?. Explor. Res. Hypothesis Med..

[166] Mulcahy J., DeLaRosby A., Norwood T. (2025). Transforming Care: Implications of Glucagon Like Peptide-1 Receptor Agonists on Physical Therapist Practice. Phys. Ther..

[167] Bliddal H., Bays H., Czernichow S., Hemmingsson J.U., Hjelmesæth J., Morville T.H., Bliddal H., Bays H., Czernichow S., Hemmingsson J.U. (2024). Semaglutide 2.4 mg efficacy and safety in people with obesity and knee osteoarthritis: Results: From the STEP 9 randomised clinical trial. Osteoarthr. Cartil..

[168] Yang Y., Hao C., Jiao T., Yang Z., Li H., Zhang Y., Shi D., Wang X., Liu Y., Huang K. (2025). Osteoarthritis treatment via the GLP-1–mediated gut-joint axis targets intestinal FXR signaling. Science.

[169] Peng W., Zhou R., Sun Z.F., Long J.W., Gong Y.Q. (2022). Novel Insights into the Roles and Mechanisms of GLP-1 Receptor Agonists against Aging-Related Diseases. Aging Dis..

[170] Najm A., Niculescu A.G., Grumezescu A.M., Beuran M. (2024). Emerging Therapeutic Strategies in Sarcopenia: An Updated Review on Pathogenesis and Treatment Advances. Int. J. Mol. Sci..

[171] Alnaser R.I., Alassaf F.A., Abed M.N. (2024). Incretin-Based Therapies: A Promising Approach for Modulating Oxidative Stress and Insulin Resistance in Sarcopenia. J. Bone Metab..

[172] Lisco G., Disoteo O.E., De Tullio A., De Geronimo V., Giagulli V.A., Monzani F., Jirillo E., Cozzi R., Guastamacchia E., De Pergola G. (2023). Sarcopenia and Diabetes: A Detrimental Liaison of Advancing Age. Nutrients.

[173] Linge J., Birkenfeld A.L., Neeland I.J. (2024). Muscle Mass and Glucagon-Like Peptide-1 Receptor Agonists: Adaptive or Maladaptive Response to Weight Loss?. Circulation.

[174] Prado C.M., Phillips S.M., Gonzalez M.C., Heymsfield S.B. (2024). Muscle matters: The effects of medically induced weight loss on skeletal muscle. Lancet Diabetes Endocrinol..

[175] Neeland I.J., Linge J., Birkenfeld A.L. (2024). Changes in lean body mass with glucagon-like peptide-1-based therapies and mitigation strategies. Diabetes Obes. Metab..

[176] Lee J.M., Sharifi M., Oshman L., Griauzde D.H., Chua K.P. (2024). Dispensing of Glucagon-Like Peptide-1 Receptor Agonists to Adolescents and Young Adults, 2020–2023. JAMA.

[177] Miller M.G., Terebuh P., Kaelber D.C., Xu R., Davis P.B. (2024). Characterizing GLP-1 Receptor Agonist Use in Preadolescent and Adolescent Populations. JAMA Netw. Open.

[178] Weghuber D., Barrett T., Barrientos-Pérez M., Gies I., Hesse D., Jeppesen O.K., Kelly A.S., Mastrandrea L.D., Sørrig R., Arslanian S. (2022). Once-Weekly Semaglutide in Adolescents with Obesity. N. Engl. J. Med..

[179] Cornejo-Estrada A., Nieto-Rodríguez C., León-Figueroa D.A., Moreno-Ramos E., Cabanillas-Ramirez C., Barboza J.J. (2023). Efficacy of Liraglutide in Obesity in Children and Adolescents: Systematic Review and Meta-Analysis of Randomized Controlled Trials. Children.

[180] Stefater-Richards M.A., Jhe G., Zhang Y.J. (2025). GLP-1 Receptor Agonists in Pediatric and Adolescent Obesity. Pediatrics.

[181] Eli Lilly and Company (2024). Open Letter Regarding the Use of Mounjaro® (Tirzepatide) and Zepbound® (Tirzepatide) [Internet]. https://lilly.gcs-web.com/news-releases/news-release-details/open-letter-regarding-use-mounjaror-tirzepatide-and-zepboundr#:~:text=Mounjaro%20and%20Zepbound%20are%20Not,%E2%80%9D)%20to%20any%20compounding%20pharmacies.

[182] Hampl S.E., Hassink S.G., Skinner A.C., Armstrong S.C., Barlow S.E., Bolling C.F., Avila Edwards K.C., Eneli I., Hamre R., Joseph M.M. (2023). Clinical Practice Guideline for the Evaluation and Treatment of Children and Adolescents with Obesity. Pediatrics.

[183] Sun R., Srivastava A., Derebail V.K., Han J., Molokie R.E., Gordeuk V., Saraf S.L. (2024). GLP-1 agonists and SGLT-2 inhibitors in adults with sickle cell disease. Am. J. Hematol..

[184] Ashruf O.S., Hundal J., Mushtaq A., Kaelber D.C., Anwer F., Singh A. (2025). Hematologic Cancers Among Patients with Type 2 Diabetes Prescribed GLP-1 Receptor Agonists. JAMA Netw. Open.

[185] Kupnicka P., Król M., Żychowska J., Łagowski R., Prajwos E., Surówka A., Chlubek D. (2024). GLP-1 Receptor Agonists: A Promising Therapy for Modern Lifestyle Diseases with Unforeseen Challenges. Pharmaceuticals.

[186] Capuccio S., Scilletta S., La Rocca F., Miano N., Di Marco M., Bosco G., Barbagallo F.G., Scicali R., Piro S., Pino A. (2024). Implications of GLP-1 Receptor Agonist on Thyroid Function: A Literature Review of Its Effects on Thyroid Volume, Risk of Cancer, Functionality and TSH Levels. Biomolecules.

[187] Lin A., Ding Y., Li Z., Jiang A., Liu Z., Wong H.Z.H., Cheng Q., Zhang J., Luo P. (2025). Glucagon-like peptide 1 receptor agonists and cancer risk: Advancing precision medicine through mechanistic understanding and clinical evidence. Biomark. Res..

[188] Baxter S.M., Lund L.C., Andersen J.H., Brix T.H., Hegedüs L., Hsieh M.H.C., Su C.T.T., Cheng M.C.Y., Chang Z.C.J., Lai E.C.C. (2025). Glucagon-Like Peptide 1 Receptor Agonists and Risk of Thyroid Cancer: An International Multisite Cohort Study. Thyroid®.

[189] Abi Zeid Daou C., Aboul Hosn O., Ghzayel L., Mourad M. (2025). Exploring Connections Between Weight-Loss Medications and Thyroid Cancer: A Look at the fda Adverse Event Reporting System Database. Endocrinol. Diabetes Metab..

[190] Balachandra S., Syed R., Song Z., Kasmirski J., Gillis A., Fazendin J., Lindeman B., Chen H. (2025). Evaluating Thyroid Cancer Risk in Glucagon-like Peptide-1 Analog Users with Thyroid Nodules. J. Surg. Res..

[191] Gravina A.G., Pellegrino R., Izzo M., De Costanzo I., Imperio G., Landa F., Tambaro A., Federico A. (2025). Relevance of Glucagon-Like Peptide 1 (GLP-1) in Inflammatory Bowel Diseases: A Narrative Review. Curr. Issues Mol. Biol..

[192] Sehgal P., Lewis J.D., Pickett-Blakely O., Nandi N., Bewtra M., Lichtenstein G.R. (2025). Safety and Clinical Effectiveness of GLP1 Receptor Agonists in Inflammatory Bowel Disease Patients. Clin. Gastroenterol. Hepatol..

[193] Marquez-Meneses J.D., Olaya-Bonilla S.A., Barrera-Carreño S., Tibaduiza-Arévalo L.C., Forero-Cárdenas S., Carrillo-Vaca L., Rojas-Rodríguez L.C., Calderon-Ospina C.A., Rodríguez-Quintana J. (2025). GLP-1 Analogues in the Neurobiology of Addiction: Translational Insights and Therapeutic Perspectives. Int. J. Mol. Sci..

[194] Qeadan F., McCunn A., Tingey B. (2025). The association between glucose-dependent insulinotropic polypeptide and/or glucagon-like peptide-1 receptor agonist prescriptions and substance-related outcomes in patients with opioid and alcohol use disorders: A real-world data analysis. Addiction.

[195] Lähteenvuo M., Tiihonen J., Solismaa A., Tanskanen A., Mittendorfer-Rutz E., Taipale H. (2025). Repurposing Semaglutide and Liraglutide for Alcohol Use Disorder. JAMA Psychiatry.

[196] Hendershot C.S., Bremmer M.P., Paladino M.B., Kostantinis G., Gilmore T.A., Sullivan N.R., Tow A.C., Dermody S.S., Prince M.A., Jordan R. (2025). Once-Weekly Semaglutide in Adults with Alcohol Use Disorder. JAMA Psychiatry.

[197] De Giorgi R., Ghenciulescu A., Dziwisz O., Taquet M., Adler A.I., Koychev I., Upthegrove R., Solmi M., McCutcheon R., Pillinger T. (2025). An analysis on the role of glucagon-like peptide-1 receptor agonists in cognitive and mental health disorders. Nat. Ment. Health.

[198] Gunturu S. (2024). The Potential Role of GLP-1 Agonists in Psychiatric Disorders: A Paradigm Shift in Mental Health Treatment. Indian J. Psychol. Med..

[199] Chen X., Zhao P., Wang W., Guo L., Pan Q. (2024). The Antidepressant Effects of GLP-1 Receptor Agonists: A Systematic Review and Meta-Analysis. Am. J. Geriatr. Psychiatry.

[200] Do D., Lee T., Inneh A., Patel U. (2025). Glucagon-Like Peptide-1 Use and Healthcare Resource Utilization for Depression and Anxiety Among Adults with Type 2 Diabetes: 2019 to 2023. J. Behav. Health Serv. Res..

[201] Ghusn W., Hurtado M.D. (2024). Glucagon-like Receptor-1 agonists for obesity: Weight loss outcomes, tolerability, side effects, and risks. Obes. Pillars.

[202] Kim J.A., Yoo H.J. (2025). Exploring the Side Effects of GLP-1 Receptor Agonist: To Ensure Its Optimal Positioning. Diabetes Metab. J..

[203] Stevens H., de la Paz M., Cooper B., Bhattacharya R. (2024). Long-term use of semaglutide and risk of diabetic retinopathy progression. Endocr. Metab. Sci..

